# Disease-Related Growth Factor and Embryonic Signaling Pathways Modulate an Enhancer of *TCF21* Expression at the 6q23.2 Coronary Heart Disease Locus

**DOI:** 10.1371/journal.pgen.1003652

**Published:** 2013-07-18

**Authors:** Clint L. Miller, D. Ryan Anderson, Ramendra K. Kundu, Azad Raiesdana, Sylvia T. Nürnberg, Roxanne Diaz, Karen Cheng, Nicholas J. Leeper, Chung-Hsing Chen, I-Shou Chang, Eric E. Schadt, Chao Agnes Hsiung, Themistocles L. Assimes, Thomas Quertermous

**Affiliations:** 1 Department of Medicine, Division of Cardiovascular Medicine, and Cardiovascular Institute, Stanford University School of Medicine, Stanford, California, United States of America; 2 Department of Surgery, Division of Vascular Surgery, Stanford University School of Medicine, Stanford, California, United States of America; 3 Division of Biostatistics and Bioinformatics, National Health Research Institutes, Zhunan, Taiwan; 4 Institute for Genomics and Multiscale Biology, Mount Sinai School of Medicine, New York, New York, United States of America; Georgia Institute of Technology, United States of America

## Abstract

Coronary heart disease (CHD) is the leading cause of mortality in both developed and developing countries worldwide. Genome-wide association studies (GWAS) have now identified 46 independent susceptibility loci for CHD, however, the biological and disease-relevant mechanisms for these associations remain elusive. The large-scale meta-analysis of GWAS recently identified in Caucasians a CHD-associated locus at chromosome 6q23.2, a region containing the transcription factor *TCF21* gene. TCF21 (Capsulin/Pod1/Epicardin) is a member of the basic-helix-loop-helix (bHLH) transcription factor family, and regulates cell fate decisions and differentiation in the developing coronary vasculature. Herein, we characterize a *cis*-regulatory mechanism by which the lead polymorphism rs12190287 disrupts an atypical activator protein 1 (AP-1) element, as demonstrated by allele-specific transcriptional regulation, transcription factor binding, and chromatin organization, leading to altered *TCF21* expression. Further, this element is shown to mediate signaling through platelet-derived growth factor receptor beta (PDGFR-β) and Wilms tumor 1 (WT1) pathways. A second disease allele identified in East Asians also appears to disrupt an AP-1-like element. Thus, both disease-related growth factor and embryonic signaling pathways may regulate CHD risk through two independent alleles at *TCF21*.

## Author Summary

As much as half of the risk of developing coronary heart disease is genetically predetermined. Genome-wide association studies in human populations have now uncovered multiple sites of common genetic variation associated with heart disease. However, the biological mechanisms responsible for linking the disease associations with changes in gene expression are still underexplored. One of these variants occurs within the vascular developmental factor, *TCF21*, leading to dysregulated gene expression. Using various *in silico* and molecular approaches, we identify an intricate allele-specific regulatory mechanism underlying altered expression of *TCF21*. Notably, we observe that two apparently independent risk alleles identified in distinct populations function through a similar regulatory mechanism. Together these data suggest that conserved upstream pathways may organize the complex genetic etiology of coronary heart disease and potentially lead to new treatment opportunities.

## Introduction

A recent meta-analysis of 14 Genome-wide association studies (GWAS) for CHD, Coronary ARtery DIsease Genome-wide Replication And Meta-analysis (CARDIoGRAM), including 22,233 cases and 64,762 controls in Europeans, elucidated 13 novel susceptibility loci [1]. One of these novel loci includes a variant, rs12190287 at 6q23.2, located within the 3′ untranslated region (3′UTR) of *TCF21* [1]. This lead SNP at 6q23.2 had the lowest *P* value (*P*<4.6×10^−11^) of the novel loci in the meta-analysis and was also highly associated in the combined meta-analysis (*P*<1.1×10^−12^). rs12190287 was also identified as an expression quantitative trait locus (eQTL) through correlation with increased *TCF21* gene expression in both liver and adipose tissue [1], [2]. Importantly, the *TCF21* locus was recently replicated in another GWAS for CHD in a Han Chinese population (15,460 cases and 11,472 controls), however a second variant (rs12524865) that is poorly correlated with rs12190287 and located 14 kb upstream of *TCF21* was the lead SNP in this racial ethnic group [3].

TCF21 is a member of the basic helix-loop-helix (bHLH) transcription factor (TF) family and regulates cell differentiation and cell fate decisions during development of the coronary vasculature, lung, kidney, and spleen [4], [5]. *Tcf21* is expressed in mesodermal cells in the proepicardial organ (PEO) as early as E9.5 in mice, and later in mesenchymal cells forming the pericardial layer [4]. Recent elegant studies employing knockout mice have established a specific role for this factor in the origin of coronary artery smooth muscle cells and cardiac fibroblasts [6], [7]. Loss of Tcf21 expression in mouse results in increased expression of SMC markers in cells on the heart surface consistent with premature SMC differentiation [7], and a dramatic failure of cardiac fibroblast development [6], [7]. These data are most consistent with a role for Tcf21 in a bipotential precursor cell for SMC and cardiac fibroblast lineages, with loss of Tcf21 expression being essential for SMC development, and persistent Tcf21 expression being required for cardiac fibroblast development [6], [7].

In studies described here we examine the function of a regulatory element at the lead variant rs12190287 though allele-specific reporter assays, gel mobility shift assays, and haplotype specific chromatin immunoprecipitation (haploChIP). We further demonstrate allele-specific regulation of *TCF21* gene expression, though modulating this regulatory element, via platelet derived growth factor receptor beta (PDGFR-β) and Wilms tumor 1 (WT1) mediated signaling. Lastly, we identify a conserved AP-1 dependent mechanism acting upstream of *TCF21* at rs12524865, which was recently associated with CHD in East Asians [3]. Taken together, these studies elucidate both disease-related and embryonic pathways upstream of *TCF21*, at two independent risk alleles, thus providing further pathophysiological insight into the common heritable risk of CHD.

## Results

### Haplotype and regulatory analysis of *TCF21* locus at 6q23.2

**Figure 1:**
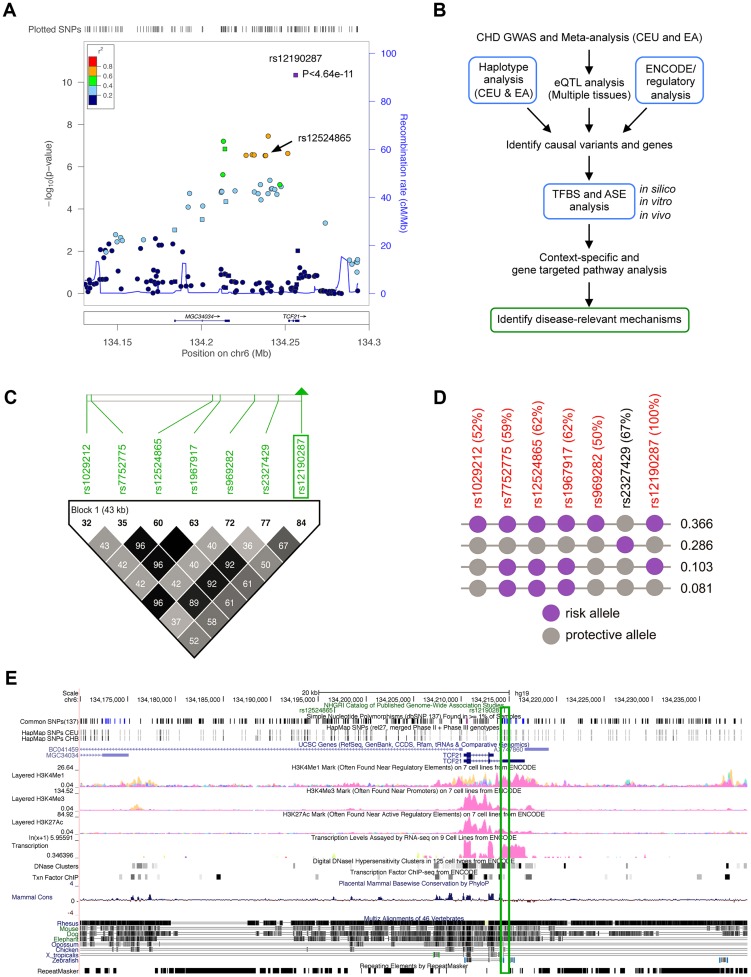
Haplotype and regulatory analysis of *TCF21* locus at 6q23.2. (**a**) Regional association plot of *TCF21* locus showing results from CARDIoGRAM meta-analysis in Caucasians, identifying rs12190287, and also depicting rs12524865. (**b**) Integrated workflow to identify CHD risk-associated mechanisms, with emphasis of approaches used in the study (blue boxes). CEU: Caucasians of European descent from UT, USA; EA: East Asians; eQTL: Expression quantitative trait loci; ASE: Allele-specific expression, TFBS: Transcription factor binding site. (**c**) Linkage disequilibrium (LD) plot of the *TCF21* locus at 6q23.2 from 1568 Metabochip genotyped Europeans (ADVANCE replication cohort) with imputation from HapMap Phase II and III CEU, showing eSNPs for *TCF21*, and rs2327429. White to black squares represent increasing r^2^ values. (**d**) The risk haplotype block containing all of the eSNPS (in red) at the *TCF21* locus has a combined frequency of 0.366. r^2^ values in LD with rs12190287 shown as a percentage in parentheses. Risk and protective alleles determined from CARDIoGRAM meta-analysis phenotypic data. (**e**) ENCODE ChIP-seq active chromatin histone modification data from 7 cell lines including promoter and enhancer marks, H3K4me1, H3K4me3, and H3K27ac. ENCODE tracks for DNase hypersensitivity, RNA-seq, and TF binding ChIP-seq data. Green box surrounds peaks overlapping rs12190287.

The CARDIoGRAM meta-analysis in Caucasians identified rs12190287 as the lead CHD association at 6q23.2 (P<4.64×10^−11^), which was 3 orders of magnitude more significant than other SNPs in this region (Figure 1A) [1]. We then set out to identify potential causal risk-associated mechanisms at 6q23.2 using an integrated workflow (Figure 1B). We examined the overall linkage disequilibrium (LD) plot around the *TCF21* locus at 6q23.2 using 1568 individuals of European descent genotyped on the fine-mapping Metabochip array [8] (Illumina), which contains 196,726 polymorphisms [8], [9], of which approximately 280 markers were contained in the 170 kb block between Chr6: 134,171,000–134,341,000. We identified regions of high LD surrounding the lead SNP, rs12190287, which is located in the 3′UTR of the non-coding exon of the long variant 1 of *TCF21*. Two haplotypes contained the high-risk allele at the lead SNP rs12190287, with one containing 3 out of the 5 additional eSNPs, while the haplotype with frequency 0.366 contained all of the eSNPS in the *TCF21* locus (Figure 1C and 1D). We examined the LD plot containing the eSNPs for *TCF21* (Figure 1C) and found that none of these variants had r^2^ values >0.8 with the lead SNP rs12190287, suggesting that if a single variant is responsible for the association observed by CARDIoGRAM, it is most likely to be rs12190287. This LD pattern is also consistent with the observation that rs12190287 was 3 orders of magnitude more significant than other SNPs in the region [1]. We then mapped regulatory chromatin regions surrounding rs12190287 using the ENCylopedia Of DNA Elements (ENCODE) Integrated Regulation data from 7 cell lines (Figure 1E), which demonstrated enrichment of the enhancer mark H3K4me1. Enrichment for histone modifications H3K4me3 (marks promoters), and H3K27ac (marks active regulatory elements) were also observed to a lesser extent. We also found regions of DNaseI hypersensitivity for open chromatin and overlapping RNA-seq peaks for transcriptional activity in the region containing rs12190287. We validated these histone modification and DNaseI ENCODE data with our own ChIP-seq and FAIRE-seq (Formaldehyde-Assisted Isolation of Regulatory Elements) experiments in HCASMC (Figure S1), demonstrating consistent regulation at rs12190287 and relevance to CHD. We also mapped the transcription factor binding sites (TFBS) using ENCODE data, which identified enrichment of an activator protein 1 (AP-1) component, JUND in a human embryonic stem cell line (Figure 1E).

### Allele-specific transcriptional regulatory activity at rs12190287 *in vitro*

**Figure 2:**
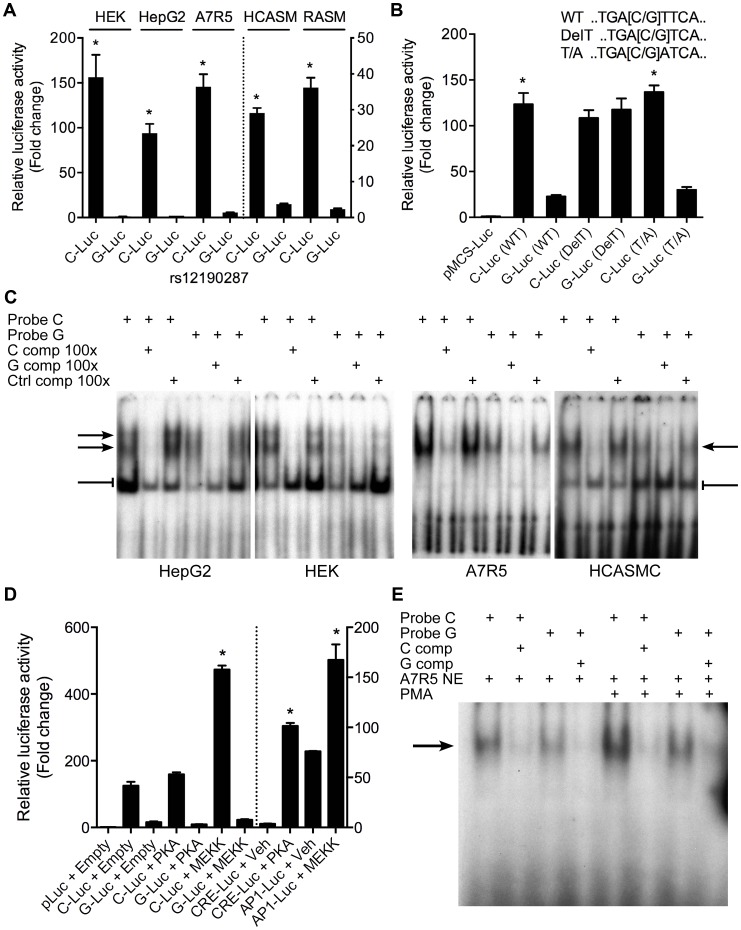
Allele-specific transcriptional activity at rs12190287. (**a**) Transcriptional activity of rs12190287-C and G variants were determined in heterozygous primary human coronary artery smooth muscle (HCASM), rat aortic smooth muscle cells (RASM) and HEK, HepG2, and A7r5 cell lines. pLuc-MCS vector containing the putative enhancer region for each rs12190287 variant (C-Luc and G-Luc) was transfected for 24 hours and the ratio of firefly and *Renilla* luciferase activities were normalized to empty reporter (pLuc). *P<0.01 versus G-Luc for each condition. (**b**) Dual-luciferase assay of wildtype rs12190287 enhancer or mutants, DelT or T/A (as shown), transfected in A7r5 as described above. *P<0.001 versus G-Luc for each condition. (**c**) Electrophoretic mobility shift assays (EMSA) showing protein binding to [γ^32^P]ATP-labeled rs12190287 C/G probes incubated with nuclear extract (NE) from various cell types, along with 100× excess C, G, or negative control (C, G, or Ctrl comp) unlabeled probe as competitor. Arrows and bar-headed lines represent specific and non-specific shifted complexes, respectively. (**d**) Dual-luciferase assay of rs12190287 C/G enhancer co-transfected with empty vector (Empty) or constitutively active protein kinase A (PKA) or mitogen-activated protein kinase (MEKK) in A7r5 using consensus CRE and AP-1 reporters as positive controls. *P<0.001 versus C-Luc+Empty or CRE-Luc+Veh or AP1-Luc+Veh where indicated. (**e**) EMSA of rs12190287 C/G with respective competition showing protein binding using control A7r5 or PMA treated A7r5 NE. Values are mean ± SD from triplicates. Similar results were observed from three independent experiments.

Given that rs12192087 appeared to be the most likely causal variant at 6q23.2, we proceeded with *in vitro* functional studies to identify the risk-associated mechanisms through this variant. First, we mapped the putative TFBS *in silico* using various bioinformatics tools, including TRANSFAC, PROMO, MatInspector, and TFSearch (Table 1). Interestingly, multiple AP-1 TFs were predicted to preferentially bind to the major risk C allele, containing the binding motif, TGACTTCA (Figure S2A). Luciferase reporters containing the putative binding site for the risk and protective alleles were then transfected into various cell lines, including HepG2, HEK, and A7r5, as well as primary human coronary artery smooth muscle cells (HCASM), and rat aortic smooth muscle cells (RASM). We observed approximately 150–200 fold increase in activity with the rs12192087-C (C-Luc) reporter relative to the empty vector reporter, and this relative activity of the C-luc reporter was ∼20-fold greater than the rs12190287-G reporter (G-Luc) (Figure 2A). Similar results were observed in primary HCASM and RASM, suggesting that the G allele disrupts TF binding, leading to reduced *TCF21* transcription. Given the ubiquitous expression of AP-1 factors in various cell types, it is not surprising that cell-specific activity was not observed. Interestingly, the allele-specific difference in transcription was lost when we mutated the 8-mer binding site to create a classical AP-1 7-mer (closely resembling a TPA element), but not when we mutated to another atypical AP-1 8-mer (Figure 2B). This is consistent with predicted binding of either c-Jun homodimers or c-Jun/ATF heterodimers, rather than classical c-Jun/c-Fos AP-1 complex [10], to confer allele-specific transcriptional regulation. In order to measure relative TF protein binding we performed electrophoretic mobility shift assays (EMSA). We observed binding to both radiolabeled alleles containing a single putative binding site in nuclear extracts from various cell types (Figure 2C). Greater binding to the C risk allele was observed, while competition with excess cold probe was more effective at the G allele. These results are consistent with the reporter assays, suggesting the G allele has weaker transcriptional regulatory activity due to decreased TF binding.

**Table 1:** *In silico* allele-specific transcription factor binding to rs12190287.

SNP	Allele	Program	Transcription factor	Sequence	Strand	Score
rs12190287	C	PROMO	JunD	TGA**C**TTC	+	98.4
			ATF3	TGA**C**TTCA	+	96.3
			c-Fos	TGA**C**TTC	+	95.9
			c-Jun	TGA**C**TTC	+	95.7
			CREM-alpha	TCGGTGA**C**TTCA	+	90.5
		TRANSFAC	ATF3	cggTGA**C**Ttcatcc	+	90.1
			ATF3	cggtga**C**TTCAtcc	−	90.0
			MEQ:cJun	ggtgA**C**TTCat	+	86.8
			MEQ:cJun	gtGA**C**TTcatc	−	85.7
		MatInspector	E4F	tcggtgA**C**TTcat	−	89.3
			Jundm2	cttcggtgA**C**TTcatccacct	+	87.5
			ATF1	acttcggtgA**C**TTcatccacc	−	87.1
		TFSearch	AP-1	GGTGA**C**TTCA	+	92.0
			CRE-BP/ATF2	TGA**C**TTCA	+	87.2
			CREB	GGTGA**C**TTCAT	+	87.0
			ATF	CGGTGA**C**TTCATC	+	86.5
		JASPAR	CREB1	TGA**C**TTCA	−	86.9
	G	PROMO	CAR:RXR-alpha	GGTGA**G**TTCA	+	95.8
			RAR-gamma	GGTGA**G**TT	+	95.4
			WT1	GTGA**G**TT	+	93.9
			PXR-1:RXR-alpha	GGTGA**G**TTCAT	+	93.9
			TFII-I	**G**TTCATCCACC	+	89.3
			c-Myb	GTGA**G**TTC	+	88.5
		MatInspector	TLX1	agaacttCGGTga**g**ttcat	+	87.3
		TFSearch	deltaE	GGTGA**G**TTCA	+	80.8
			c-Myb	**G**TTCATCCA	+	79.4
			AP-1	GGTGA**G**TTCA	+	77.9
			C/EBP	ACTTCGGTGA**G**T	+	76.9
		JASPAR	CREB1	TGA**G**TTCA	+	79.8

Predicted transcription factor binding site searches were performed using five programs: TRANSFAC, PROMO, MatInspector, TFSearch, and JASPAR. The SNP is shown in boldface within the predicted binding sequence. Core binding sequence shown in capital letters for TRANSFAC and MatInspector generated sequences. The scores represent matrix sequence similarity for each program. A minimum threshold of 85.0 was used for each program, and lowered to 75.0 for allelic comparison if necessary.

### AP-1-like transcription factor binding to rs12190287 *in vitro*

Given that the putative binding site closely resembles an AP-1 or CRE element, we measured the effects of activating protein kinase C (PKC) via phorbol-12-myristate-13-acetate (PMA) or adenylyl cyclase (AC) via forksolin (fsk) on the transcriptional activity at rs12190287 (Figure S2B). Surprisingly, neither PMA nor forskolin altered C or G-Luc reporter activity, while both activated the consensus AP-1 and CRE reporters, respectively. This may indicate the element at rs12190287 can be activated in normal growth media, which contains growth factors upstream of AP-1. Overexpression of constitutively active MEKK (preferentially upstream of AP-1 elements), but not active protein kinase A (PKA) (preferentially upstream of CRE elements) led to greater transactivation of the C allele (Figure 2D). We observed an increase in the bound TF complex upon PMA stimulation, which was greater at the C allele, suggesting binding of an AP-1-like element (Figure 2E). This is further supported by the gel shift observation of a similar higher molecular weight complex bound to C and G alleles, compared to the consensus AP-1 probe (Figure S2C). Interestingly, a second lower molecular weight complex bound to the consensus AP-1 probe was not observed with C and G probes, suggesting some differences in TFs binding to these distinct elements.

### AP-1 dependent transcriptional regulation at rs12190287 *in vitro*

**Figure 3:**
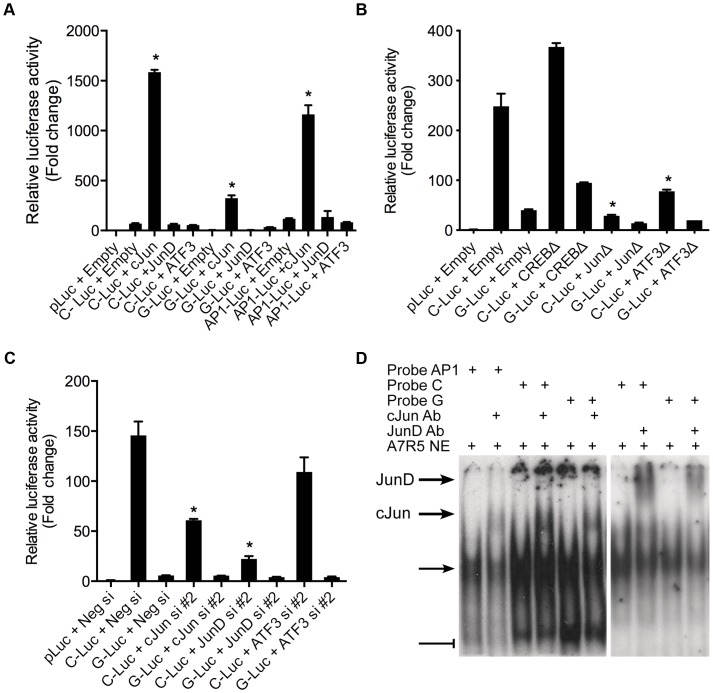
AP-1 dependent transcriptional regulation at rs12190287 *in vitro*. (**a**) Dual-luciferase assay of rs12190287-C and G variants determined in A7r5 cells transfected with rs12190287 enhancer (C-Luc and G-Luc), consensus AP-1 reporter (AP1-Luc) or empty reporter (pLuc), along with human c-JUN, JUND, or ATF3 expression vectors and measured after 24 hours. *P<0.0001 versus C-Luc, G-Luc or −1-Luc+Empty where indicated. (**b**) Dual-luciferase assay of rs12190287 enhancer in A7r5 cells transfected with empty vector (Empty) or dominant negative expression constructs for human CREB (CREBΔ), c-JUN (JunΔ), or ATF3 (ATF3Δ). *P<0.01 versus C-Luc+Empty. (**c**) Dual-luciferase assay of rs12192087 enhancer in heterozygous HCASMC transfected with small interfering RNA duplexes (siRNA) against c-*JUN*, *JUND*, *ATF3* or a negative control (Neg si). *P<0.01 versus C-Luc or G-Luc+Neg si. (**d**) EMSA of rs12190287 C/G enhancer showing specific shifted complexes (arrow) and JunD and c-Jun super-shifted complexes (bold arrows). Bar-head line denotes non-specific shifted complexes. Values are normalized mean ± SD from triplicates. Similar results were observed from three independent experiments.

We then sought to determine the specific identity of the TFs predicted to bind rs12192087 *in vitro*. Using allele specific reporters for rs12190287, we measured the regulatory effects of overexpression of AP-1 related factors meeting a predicted *in silico* binding threshold of >0.85, which included c-Jun, JunD, and ATF3 (Figure 3A). c-JUN overexpression elicited robust activation of the C allele, similar to the activation of consensus AP-1-luc. Less overall activity was observed with the G allele and minor effects were observed upon JUND and ATF3 overexpression. Prior reports also demonstrate that c-Jun predominately activates AP-1 elements *in vitro*, whereas JunD and ATF3 alone often result in transrepression [11]. We also measured the transcriptional regulation at rs12192087 via loss-of-function experiments. The dominant negative mutant ΔJun (TAM67), which lacks the transactivation domain of c-Jun, resulted in blunted transcriptional activity at C and G alleles (Figure 3B). Similar results were observed with ΔATF3, whereas ΔCREB led to slightly increased activity. Transfection of siRNAs against c-*JUN*, *JUND*, and *ATF3* also led to reduced transcriptional activity at the C and G alleles, specifically implicating these factors in mediating the activity at rs12190287 (Figure 3C). siRNA-mediated protein knockdown for each AP-1 TF was confirmed by immunoblotting (Figure S3). Using EMSA we also observed super-shifted complexes upon incubation with antibodies against c-Jun and JunD (Figure 3D). Together these results implicate the AP-1 factors c-Jun, JunD, as well as ATF3 in regulating putative enhancer activity at rs12190287.

### PDGFRβ is upstream of *TCF21* at rs12190287 in HCASMC

**Figure 4:**
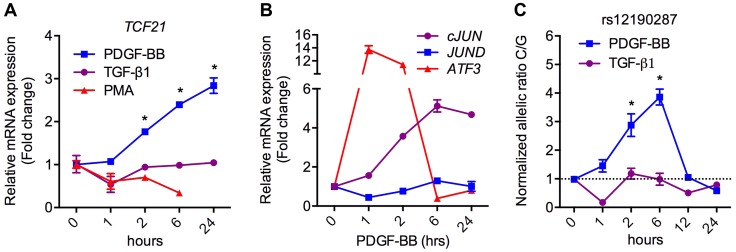
PDGFRβ is upstream of *TCF21* at rs12190287 in HCASMC. (**a**) TaqMan based qRT-PCR results showing relative mRNA expression levels of total *TCF21* in heterozygous HCASMC treated with TGF-β1, PDGF-BB or PMA for indicated times. Expression levels were normalized to 18S and expressed as fold change from 0 h. *P<0.0001 versus 0 h. (**b**) TaqMan qRT-PCR expression results of c-*JUN*, *JUND*, and *ATF3* in heterozygous HCASMC treated TGF-β1 or PDGF-BB for the indicated times. (**c**) Allele-specific TaqMan qRT-PCR based expression of *TCF21* at rs12190287 shown as normalized allelic ratio C/G in heterozygous HCASMC treated with either TGF-β1 or PDGF-BB for the indicated times. *P<0.01 versus 0 h. Values are mean ± SD of triplicates. Similar results were observed from three independent experiments.

To ascertain the functional implications of allelic variation at rs12190287, we measured the effects of relevant upstream stimuli on *TCF21* expression in HCASMC. Platelet-derived growth factor (PDGF) is a potent growth factor ligand responsible for activation of AP-1-dependent gene expression in SMCs, leading to synthetic phenotypic properties such as increased proliferation, survival, and migration [12]. Further, signaling through PDGFRβ is required for epithelial-mesenchymal transition (EMT) and epicardial fate determination in developing CASMC [13]. Transforming growth factor beta (TGF-β1) is also critically involved in both EMT and adult SMC phenotypic modulation. Interestingly, PDGF-BB treatment resulted in a time-dependent increase in *TCF21*, whereas transforming growth factor beta (TGF-β1) and PMA led to slightly reduced or unaltered *TCF21* levels (Figure 4A). Western blots demonstrated changes in TCF21 protein levels were consistent with changes in *TCF21* mRNA levels in response to PMA and PDGF-BB (Figure S4). Concordantly, c-*JUN* and *ATF3* were upregulated by PDGF-BB, while *JUND* levels were unchanged (Figure 4B). We then assessed the effects of PDGF-BB and TGF-β1 treatment on allele-specific expression (ASE) of *TCF21* in HCASMC using TaqMan allelic discrimination (Figure 4C, Figure S5A, B). Using heterozygous CG HCASMC, we observed that PDGF-BB treated samples had greater normalized C/G ratios, which peaked around 6 hours, while TGF-β1 treated samples had lower C/G ratios (Figure 4C). The phasic allelic imbalance observed with PDGF-BB may be partially dependent on activation of AP-1 to regulate *TCF21* gene expression.

### AP-1 mediated regulation at rs12190287 *in vivo*

**Figure 5:**
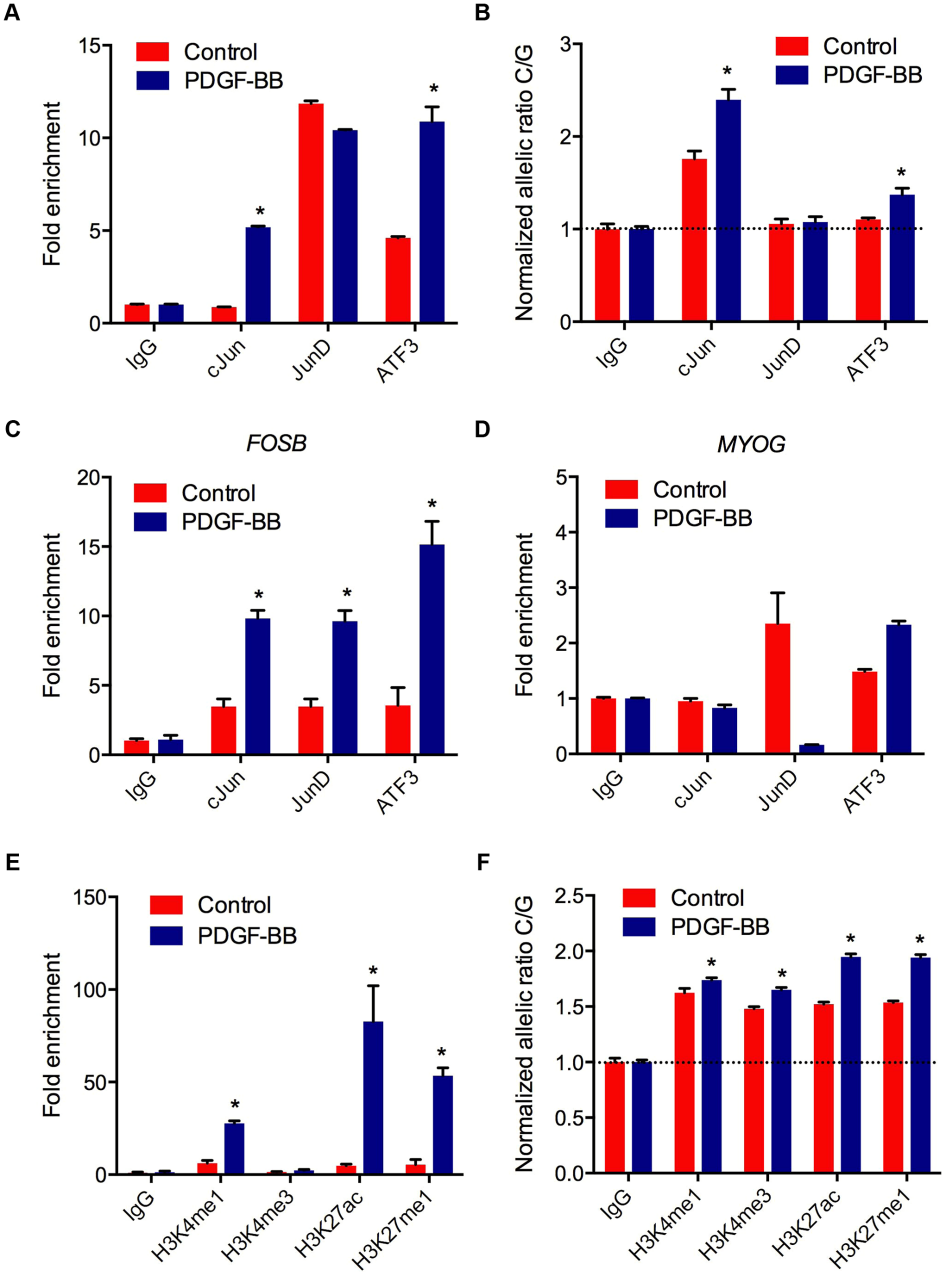
AP-1 mediated regulation at rs12190287 *in vivo*. (**a**) Total enrichment of c-Jun, JunD, and ATF3 at rs12190287 determined by chromatin immunoprecipitation (ChIP) in heterozygous HCASMC treated with PDGF-BB (20 ng/ml) or vehicle (Control) for 6 hrs. (**b**) Allele-specific enrichment of c-Jun, JunD and ATF3 at rs12190287 determined by HaploChIP in heterozygous HCASMC treated with PDGF-BB, shown as normalized allelic-ratio C/G. (**c**–**d**) AP-1 positive and negative control regions for c-Jun, JunD, and ATF3 enrichment at *FOSB* (**c**) and *MYOG* (**d**) promoters, respectively. (**e**) Total enrichment of histone modifications H3K4me1, H3K4me3, H3K27ac, and H3K27me1 at rs12190287 determined by ChIP in heterozygous HCASMC treated with PDGF-BB. (**f**) Allele-specific enrichment of H3K4me1, H3K4me3, H3K27ac, and H3K27me1 at rs12190287 determined by HaploChIP in heterozygous HCASMC treated with PDGF-BB, shown as normalized allelic ratio C/G. *P<0.001 versus Control for each condition. Values are mean ± SD from triplicates. Similar results were observed from 3–4 independent experiments. For HaploChIP experiments, separate heterozygous individual HCASMCs were used for each replicate experiment.

Transcriptional regulation of gene expression is tightly controlled by the native chromatin architecture *in vivo*. Therefore, we interrogated allele-specific AP-1 occupancy at rs12190827 using chromatin immunoprecipitation (ChIP) and haplotype specific ChIP (haploChIP). In HCASMC treated with PDGF-BB we observed a significant increase in enrichment at rs12190287 by c-Jun and ATF3 (Figure 5A). JunD enrichment, while significantly above IgG in control samples, was unchanged with PDGF-BB. We then measured allele-specific enrichment in heterozygous HCASMC treated with PDGF-BB, as done previously for haploChIP [14]. Interestingly, c-Jun was predominately enriched at the C allele under control treatment, indicated by greater C/G ratios (Figure 5B). Both c-Jun and ATF3 were more enriched at the C allele upon PDGF-BB treatment, and JunD enrichment was unchanged. Similar observations were made using pyrosequencing-based allelic discrimination (Figure S5C). ChIP products were also amplified at *FOSB* and *MYOG* promoters, as AP-1 positive and negative control regions, respectively (Figure 5C, D). We then measured putative enhancer activity at rs12190287 via post-transcriptional histone modification. PDGF-BB treatment led to significantly increased enrichment of H3K4me1 (marks active/poised promoters) and H3K27ac (marks active enhancers) and H3K27me1 (marks active/poised promoters) (Figure 5E). We also observed increased relative enrichment of active histone modifications at the C allele, which was further potentiated with PDGF-BB stimulation in HCASMC (Figure 5F). These data indicate that the AP-1 complex positively regulates the rs12190287 risk allele in the native and active chromatin state.

### WT1 regulation of rs12190287 *in vitro* and *in vivo*

**Figure 6:**
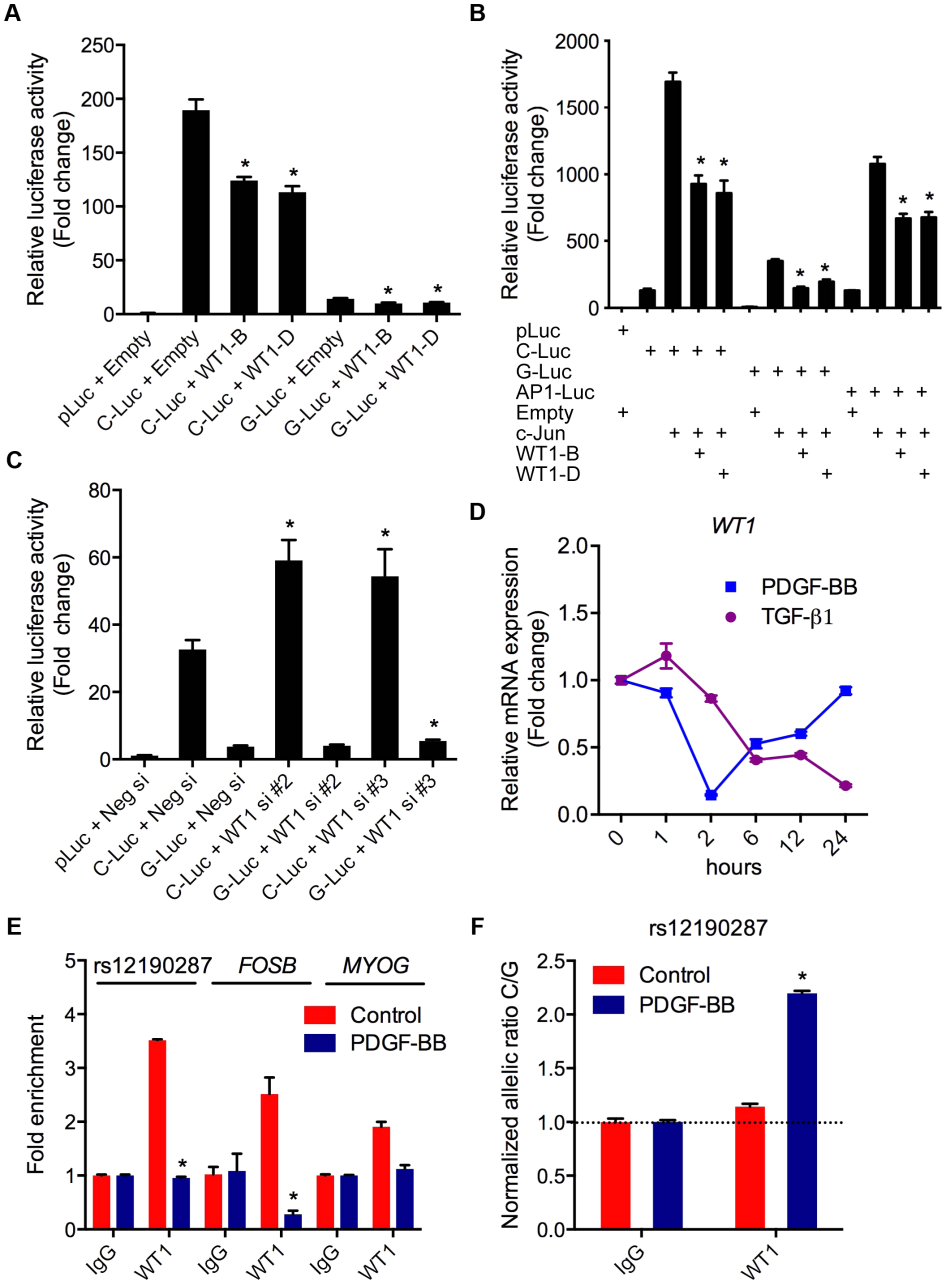
WT1 regulation of rs12190287 *in vitro* and *in vivo*. (**a**) Dual-luciferase assay of rs12190287 C/G enhancer transfected with human WT1-B (−KTS), WT1-D (+KTS) expression constructs in A7r5 cells, measured after 24 hours. *P<0.01 versus C-Luc or G-Luc+Empty. (**b**) Dual-luciferase assay of rs12190287 C/G enhancer co-transfected with human c-JUN and WT1-B or WT1-D expression constructs in A7r5 cells. AP1-Luc reporter was used as a positive control. *P<0.05 versus C-Luc or G-Luc+cJun. (**c**) Dual-luciferase assay of rs12190287 C/G enhancer transfected in heterozygous HCASMC with siRNA against *WT1* (−/+KTS) compared to negative control (Neg si). *P<0.05 versus C-Luc or G-luc+Neg si. (**d**) TaqMan based qRT-PCR results showing relative human *WT1* mRNA expression levels in heterozygous HCASMC treated with TGF-β1 or PDGF-BB for the indicated times. (**e**) Total enrichment of WT1 at rs12190287 enhancer, *FOSB* or *MYOG* promoter regions determined by chromatin immunoprecipitation (ChIP) in heterozygous HCASMC treated with PDGF-BB for 6 hrs. Values represent fold change relative to enrichment with IgG control. *P<0.01 versus Control WT1. (**f**) Allele-specific enrichment of WT1 at rs12190287 determined by HaploChIP in heterozygous HCASMC treated with PDGF-BB, shown as normalized allelic-ratio C/G. *P<0.0005 versus Control WT1. Values are mean ± SD from triplicates. Similar results were observed from three independent experiments.

We also investigated the potential functional effects of non-AP-1 TFs predicted to bind rs12190287 (Table 1). Wilms tumor 1 (WT1) was of particular interest given its known role in CASMC development [15], and evidence in developmental models indicating *wt1* directly regulates *tcf21* [16]. The G allele resides in a WT1-like binding element [17] (WTE; 5′-GCGTGGGAGT-3′), which was previously implicated in the regulation of the human thromboxane A2 receptor [18]. WT1-D (+KTS amino acid insertion) binds DNA with reduced affinity compared to WT1-B (−KTS) [19], and the ratio of the two alternatively spliced isoforms has been implicated in Frasier syndrome [20]. We observed that expression of WT1-B (−KTS) and WT1-D (+KTS) led to similar transrepression of both C and G alleles at rs12190287 (Figure 6A), consistent with the role of WT1 as a transcriptional repressor. As a tumor suppressor gene, WT1 often represses AP-1 mediated transcription [21], [22] and WT1-B and WT1-D also repressed c-Jun-mediated activation of rs12190287 *in vitro* (Figure 6B). Similar regulation was observed at both C and G alleles suggesting WT1-mediated regulation may not be the rate-limiting step altered by rs12190287. Consistently, *WT1* siRNA mediated knockdown led to increased activity of C and G alleles (Figure 6C), with protein knockdown verified by immunoblotting (Figure S3D). Next, we assessed the expression changes of *WT1* upon TGF-β1 or PDGF-BB treatment of HCASMC (Figure 6D). Interestingly, PDGF-BB led to a rapid decline in *WT1*, which recovered by 24 hours. TGF-β1 led to a slower yet persistent reduction of *WT1*. We also observed decreased enrichment of WT1 at rs12190287 upon PDGF-BB stimulation, consistent with effects observed at the *FOSB* promoter (AP-1 positive control region) (Figure 6E). Less reduction was observed at the *MYOG* promoter (AP-1 negative control region). Surprisingly we observed WT1 preferentially enriched at the C allele, which was increased upon PDGF-BB stimulation (Figure 6F). Given that WT1 negatively regulates transcription at rs12190287 and is downregulated by PDGF-BB, may suggest that the WT1 cofactor preferentially associates with the C risk allele to temporally fine-tune AP-1 mediated transcription upon growth factor stimulation.

### AP-1 regulation at rs12524865 and haplotype structure in East Asians

**Figure 7:**
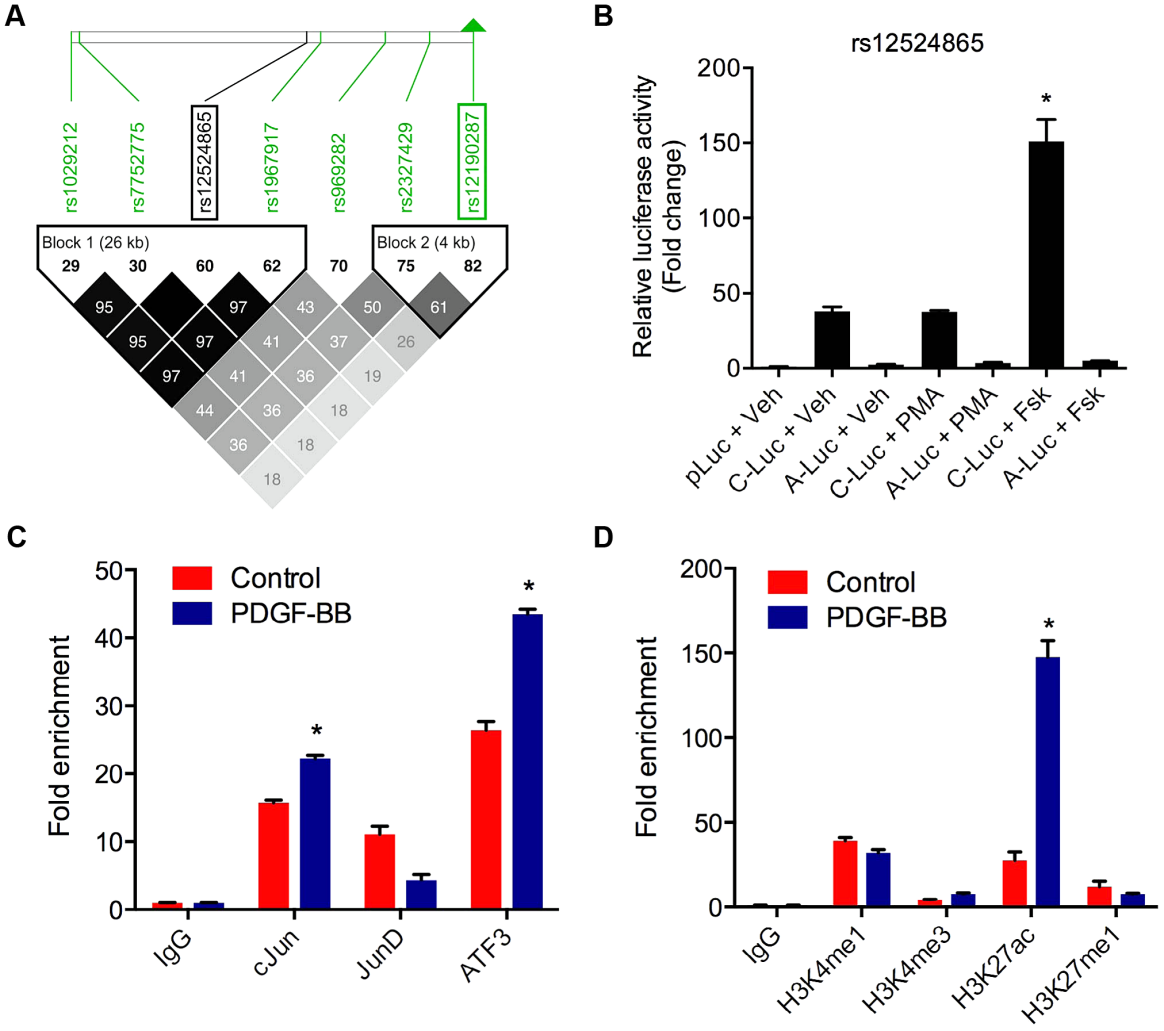
AP-1 regulation at rs12524865 and haplotype structure in East Asians. (**a**) Linkage disequilibrium (LD) plot from ∼2400 Metabochip genotyped East Asian samples from the TAICHI study (HALST cohort) with imputation from HapMap Phase II and III Han Chinese (CHB), showing eSNPs for *TCF21* and distinct haplotype blocks containing rs12524865 and rs12190287. White to black squares represent increasing r^2^ values, also shown in blocks. (**b**) Dual-luciferase assay of rs12524865 C/A enhancer transfected in A7r5 and treated with adenylyl cyclase activator, forskolin (Fsk) or PKC activator, phorbol-12-myristate-13-acetate (PMA) for 4 hours. Relative luciferase activities measured after 24 hours. *P<0.01 versus C-Luc+Veh. (**c**) Total enrichment of c-Jun, JunB, JunD, and ATF3 at rs12524865 determined by chromatin immunoprecipitation (ChIP) in HCASMC treated with PDGF-BB or vehicle (Control) for 6 hours. *P<0.005 versus Control for each condition. (**d**) Total enrichment of histone modifications H3K4me1, H3K4me3, H3K27ac, and H3K27me1 at rs12524865 determined by ChIP in HCASMC treated with PDGF-BB. *P<0.001 versus Control for each condition. Values are mean ± SD from triplicates. Similar results were observed from three independent experiments.

**Figure 8:**
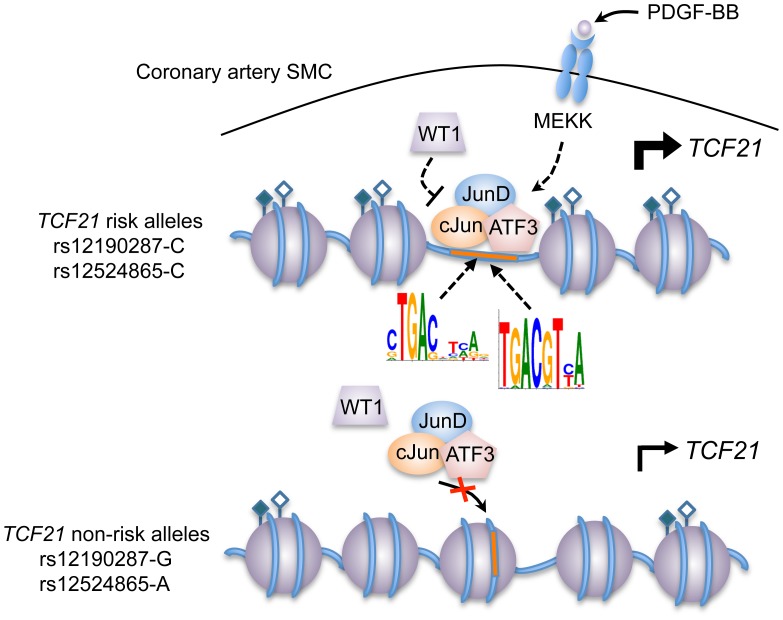
Predicted model of AP-1 dependent regulation of *TCF21* at rs12190287 and rs12524865. Individuals carrying risk alleles for rs12190287 or rs12524865 at 6q23.2 are expected to have increased *TCF21* expression upon stimulation of PDGFR-β by PDGF-BB in coronary artery smooth muscle cells, due to increased enrichment of active histone modifications (represented by closed and open diamonds) leading to an open chromatin conformation, allowing binding of an active AP-1 TF complex containing various combinations of c-Jun, JunD, and ATF3. WT1 functions as a transrepressor of this active complex at rs12190287, whereby WT1 may fine-tune the spatial and temporal activation of *TCF21* expression. Multiple kinases other than mitogen-activated kinase (MEKK) may be involved in the activation and recruitment of AP-1 complexes to these binding sites.

*TCF21* was one of three Caucasian CAD associated loci that was recently replicated in a Han Chinese population [3]. While association at rs12190287 did not reach genome-wide significance at the *TCF21* locus, rs12524865 (Figure 1A) represented the lead SNP in this racial ethnic group and showed consistent association in the discovery and replication stages [3]. We examined the haplotype structure at 6q23.2 in ∼2400 Han Chinese from the HALST study in Taiwan who were also genotyped with the Metabochip, and augmented genotype data in this region through imputation using data from the HapMap II and III Han Chinese (CHB) samples. We identified regions of high LD surrounding rs12190287, however much less LD surrounding rs12524865 compared to the European samples, and we found that rs12524865 is located in a distinct haplotype block from the lead SNP in Europeans, rs12190287 (Figure 7A). Further, the risk haplotype containing rs12524865 and four other *TCF21* eSNPs occurs at similar frequency (0.361) in this population (Figure S6A). rs12524865 is in perfect LD with other eSNPs for *TCF21* in one haplotype block, including rs1967917 and rs7752775. However, the haplotype block for rs12190287 does not contain other alleles in LD (Figure 7A). We then mapped the putative TFBS at rs12524865 using multiple prediction tools (Table 2). Interestingly, rs12524865 is also located within an AP-1/CREB-like element, TAA[C/A]GTCA, which closely resembles the consensus ATF/CREB binding site, TGACGTCA (Figure S6B). As expected, the C allele (also major, risk allele) is predicted to bind AP-1 and CREB family members, whereas predicted binding is disrupted by the minor, protective allele. Using luciferase reporters containing this putative enhancer we observed robust transcriptional activity with the risk allele, which was absent with the protective allele (Figure 7B). Forskolin but not PMA stimulation potentiated this activity, suggestive of a cAMP-responsive ATF/CREB element (Figure 7B). Dominant negative mutants of CREB, Jun, and ATF3 reduced this activity (Figure S6C). We then measured occupancy of AP-1 and active chromatin histone modifications at rs12524865. Interestingly, we observed increased c-Jun and ATF3 enrichment with PDGF-BB treatment, with much greater enrichment by ATF3 (Figure 7C). Enrichment of active histone modifications, H3K4me1 and H3K27ac also suggest a functionally active chromatin state at rs12524865 (Figure 7D). Together these results implicate both rs12190287 and rs12524865 risk alleles in a shared AP-1-dependent mechanism for regulating *TCF21* in HCASMC, thus further defining the genetic risk mechanisms of CHD which have been conserved across racial ethnic groups during evolution (Figure 8).

**Table 2:** *In silico* allele-specific transcription factor binding to rs12524865.

SNP	Allele	Program	Transcription factor	Sequence	Strand	Score
rs12524865	C	TRANSFAC	ATF2:cJun	taA**C**GTCa	−	100
			CREB1	cttaa**C**GTCAgt	+	99.1
			CREB/ATF	taa**C**GTCAg	−	99.0
			ATF2	ttaa**C**GTCAgtc	−	98.8
			CREM	taa**C**GTCAgtc	+	97.5
		PROMO	CREB	TTAA**C**GTCA	+	97.9
			ATF2	TTAA**C**GTCAG	+	95.3
			ATF1	TAA**C**GTCAGTC	+	93.7
			ATF3	TAA**C**GTCA	+	93.2
			c-Jun	AA**C**GTCA	+	91.9
		MatInspector	CREB2:cJun	aataacttaA**C**GTcagtccca	−	100
			ATF1	ataacttaA**C**GTcagtcccaa	+	84.1
			CREB	aacttaa**c**GTCAgtcccaaag	−	91.8
			E4F	aacttaA**C**GTcag	−	88.7
		TFSearch	CRE-BP	TTAA**C**GTC	+	87.5
			CREB	TTAA**C**GTC	+	86.1
		JASPAR	CREB1	TAA**C**GTCA	−	92.3
			MIZF	AA**C**GTCAGT	+	84.9
			c-Jun	AA**A**GTCA	+	94.2
	A	PROMO	ATF3	TAA**A**GTCA	+	86.5
			AP-1	TTAA**A**GTCA	+	85.9
		MatInspector	PPARG	tcagtcccAAAGgctaa**a**g	+	83.4
		TFSearch	CdxA	A**A**GTCA	+	88.5
			Nkx2.5	TTAA**A**GT	+	83.7
		JASPAR	AP-1	**A**GTCAGTCCC	+	81.4
			CRE-BP	CTTAA**A**GT	+	76.0
			CREB1	TAA**A**GTCA	−	78.7
			MIZF	TAA**A**GTCAGT	+	78.0

Predicted transcription factor binding site searches were performed using five programs: TRANSFAC, PROMO, MatInspector, TFSearch, and JASPAR. The SNP is shown in boldface within the predicted binding sequence. Core binding sequence shown in capital letters for TRANSFAC and MatInspector generated sequences. The scores represent matrix sequence similarity for each program. A minimum threshold of 85.0 was used for each program, and lowered to 75.0 for allelic comparison if necessary.

## Discussion

Therapeutic targeting of traditional CHD risk factors has reduced overall mortality rates, however there are currently no therapies that directly target disease processes of the vessel wall. Recent GWAS have identified 46 independent risk-associated loci for CHD/MI, and 104 independent loci associated at a false discovery rate <5% [8], [23]. Many of the genes identified are implicated in the regulation of SMC plasticity during atherosclerosis, including *PDGFD*, *COL4A1/2*, *CDKN2B* and *CDKN2A/p19ARF* [24]–[26]. However, the molecular mechanisms and relevant pathways underlying these risk associations are relatively underexplored. Here, using an integrated beyond GWAS strategy (Figure 1B), we reveal the interplay of both developmental and disease-related pathways, which coordinate the regulation of *TCF21* at independent CHD susceptibility alleles in humans. Individuals carrying the risk haplotype for rs12190287 and rs12524865 are predicted to have greater *TCF21* expression due to increased binding of AP-1 complexes to a *cis*-regulatory element. Our studies further reveal a potential PDGFRβ-dependent mechanism for the CHD risk association in human coronary artery smooth muscle cells both *in vitro* and *in vivo*.

Of the 13 novel loci identified in the initial CARDIoGRAM meta-analysis, *TCF21* was particularly attractive as a missing link to CHD. Tcf21 (Pod-1/Capsulin/Epicardin) was initially cloned in our laboratory and two others [4], [27], and is expressed in epicardial progenitor cells that give rise to developing CASMC [4]. Studies of TCF21 function in the adult have been hampered since *Tcf21* null mice die postnatally due to pulmonary hypoplasia and respiratory failure [5]. TCF21 has been identified as a candidate tumor suppressor gene and is frequently epigenetically silenced in various human cancers [28], [29]. These studies have implicated loss of TCF21 expression as an early-stage biomarker for increased cancer risk. Based on our findings, we can reason that aberrant upregulation of TCF21 in coronary SMC may increase CHD risk through alteration of the SMC response to injury in the vessel wall.

Identification of *cis*-regulatory elements altered by disease related variants is critical for post-GWAS functional characterization studies [30]–[32]. Here, we observed that PDGF-BB mediates binding of an AP-1 complex, likely containing c-Jun, and JunD or ATF3 heterodimers to the risk allele at rs12190287, which is preferentially in an active chromatin state. The activator protein-1 (AP-1) family of TFs have been implicated in growth factor-dependent SMC activation following vessel injury [33]. The prototypical basic region-leucine zipper (bZip) protein, c-JUN is expressed in human atherosclerotic plaques and promotes SMC proliferation and neointima formation *in vivo* [34]. ATF3 is another stress-inducible gene upregulated in many cancers [35] and also in SMCs within injured mouse femoral arteries, to promote SMC migration and ECM synthesis [36]. It has been shown that c-Jun readily forms heterodimers with ATF2 and ATF3, which have distinct DNA binding affinities to CRE and AP-1 elements [37]. Interestingly, genes encoding extracellular matrix (ECM) proteins are often the targets of c-Jun/ATF enhancer elements [10].

A challenge in prioritization of regulatory SNPs is elucidating the biologically relevant upstream pathways driving these associations [38], [39]. Platelet-derived growth factor (PDGF) is a critical growth factor involved in vascular development. It has been shown in mice that PDGFR-β is required for development of mural cells, CASMC and pericytes, involving epithelial-to-mesenchymal transition (EMT) in the epicardium [13], [40]. PDGF-BB is also a potent inducer of the synthetic SMC phenotype, increasing migration, lipid uptake, and ECM synthesis, both *in vitro* and *in vivo* during vascular injury and atherosclerosis [12]. A recent GWAS for CHD in Europeans and South Asians identified the *PDGFD* gene, and this PDGF family member has been shown to have many of the same disease-related actions as related PDGFs [41]. In contrast, TGF-β is a pleiotropic cytokine mostly responsible for maintaining SMC differentiation, through activation of Smad, SRF, and RhoA signaling pathways [42]. Indeed, VSMC differentiation during aortic development likely depends on TGF-β rather than PDGF-BB/PDGFR-β [43]. Our observations that *TCF21* was selectively induced by PDGF-BB rather than TGF-β in HCASMC is consistent with the notion that TCF21 inhibits coronary artery SMC differentiation while inducing SMC phenotypic modulation.

Similar to Tcf21, Wilms tumor 1 (Wt1) is expressed in the early proepicardium, epicardium and mesenchyme during development of the heart and other mesoderm-derived tissues [15], [44], [45]. In fact, previous studies in zebrafish have demonstrated that tcf21 expression in the proepicardial organ is dependent on wt1 [46]. Wt1 expression is also induced in the coronary vasculature in regions of ischemia and hypoxia after MI in mice [47]. As a tumor suppressor gene, *WT1* was previously shown to repress PMA induced transcription [22]. WT1 binding to the thrombospondin-1 promoter leads to repression upon c-Jun overexpression in ECs [48] and fibroblasts [21]. Here we identify WT1 upstream of *TCF21* to repress the enhancer at rs12190287. While *in silico* analyses predicted preferential binding to the G allele, haploChIP data suggest that WT1 preferentially associates with the C allele. This is consistent with greater c-Jun enrichment at the C allele. The orthogonal regulation of WT1 expression by PDGF-BB compared to *TCF21*, c-*JUN*, *JUND*, and *ATF3*, also may imply that WT1 acts to spatially and temporally fine-tune *TCF21* expression, rather than cause repression.

CHD involving atherosclerosis continues to burden both developed and developing countries, largely due to urbanization and westernization of diet and lifestyle. Compared to developed countries, CHD related deaths are predicted to rise more than 3-fold in China and India, for instance [49]. While most GWAS for CHD have focused on individuals of European ancestry, large-scale studies of non-European populations may allow further understanding of the risk-associated mechanisms driving CHD. A recent meta-analysis of GWAS for CHD in a Han Chinese population (15,460 cases and 11,472 controls) replicated the *TCF21* association in Europeans [3]. The combined discovery and replication stages identified a near genome-wide significant association at rs12524865, upstream of *TCF21* at 6q23.2 (*P* = 1.87×10^−7^). The discovery stage identified primarily rs12524865 (*P* = 3.40×10^−3^), although rs12190287 showed a trend and directionality as a reporter for Caucasian cohorts (*P* = 3.03×10^−2^). The linkage disequilibrium r^2^ values between these two eQTLs for *TCF21* is 0.62 in Europeans and only 0.18 in Han Chinese, and these variants are found in separate haplotype blocks in Han Chinese. Further, we demonstrated rs12524865 disrupts a binding site for CREB/ATF *in silico* and measured enrichment for c-Jun and ATF3 at rs12524865 in HCASMC. These studies highlight the value of multi-ethnic post-GWAS validation of causal variants to assess both the functional impact and heritable risk of common variants in complex diseases.

The compelling promise of the new disease associated genes and pathways afforded by GWAS methodology is that they will provide biological insights and targets for the development of new therapeutic approaches, and this is particularly compelling for CHD where there are no therapies directed at the blood vessel wall. TCF21 and its downstream targets provide one such pathway. eQTL data have suggested that the disease-associated major allele shows increased *TCF21* expression, and this is consistent with the functional studies described herein, where the major risk C alleles at rs12190287 and rs12524865 confer greater transcriptional activity compared to the minor protective G and A alleles, respectively. The embryonic function of TCF21 in the developing coronary circulation seems most consistent with a role aimed at inhibiting differentiation of SMC progenitors, and thus it is likely that TCF21 function might interfere with the SMC response to vascular injury in the disease setting. It is now generally accepted that vascular SMC provide a stabilization of the atherosclerotic plaque, and thus therapeutic inhibition of the TCF21 pathway would be expected to decrease the risk for coronary events. However, such an approach might also put the patient at increased risk for head and neck and lung cancer, as TCF21 is a potent tumor suppressor gene that is frequently mutated or silenced in cells of these tumors. This situation contrasts with the emerging information related to the risk mechanisms at 9p21.3, where the function of one likely causal gene *CDKN2B* is associated with decreased risk for vascular disease [25], [50] as well as a broad range of human cancers [51]–[53]. Therapeutic activation of expression of this gene would be expected to decrease risk for both types of disease. While it is still early days for such extrapolations, follow-up of vascular wall GWAS genes is expected to provide insights into disease-related pathways to better inform therapeutic manipulation.

## Materials and Methods

### Cell culture

Primary human coronary artery smooth muscle cells (HCASMC) were purchased from three different manufacturers, Lonza, PromoCell and Cell Applications and were cultured in complete smooth muscle basal media (Lonza) according to the manufacturer's instructions. All experiments were performed on HCASMC between passages 4–7, using lots identified as heterozygous at rs12190287 as indicated. Primary rat aortic smooth muscle cells (RASM) were obtained from Dr. Phil Tsao (Stanford University) and cultured in Dulbecco's' Modified Eagle Media (DMEM) low glucose with 10% fetal bovine serum (FBS). The rat aortic smooth muscle cell line, A7r5 was purchased from ATCC and also obtained from Dr. Joe Miano (University of Rochester) and were maintained in DMEM low glucose with 10% FBS. HepG2 and HEK cells were purchased from ATCC and maintained in DMEM low glucose with 10% FBS.

### siRNA transfection

Pre-designed Silencer Select siRNA duplexes against human c-*JUN*, *JUND*, *ATF3*, and *WT1* were purchased from Ambion/Life Technologies. At least two individual siRNAs were tested for each. Briefly, HCASMC were plated in 12-well (dual-luciferase assay) or 6-well plates (qPCR) in complete media. Approximately 24 hours after plating, and between 40–60% confluence, cells were transiently transfected with negative control or TF specific siRNAs (50 nM) using RNAiMAX reagent (Life Technologies) according to the manufacturer's instructions. Cells were incubated for 48 hours prior to performing dual-luciferase assays, harvesting total RNA using miRNeasy Mini kit (Qiagen) for TaqMan based qPCR assays, or nuclear protein extraction for Western blotting.

### Dual-luciferase assay

Oligonucleotides containing the putative enhancer elements for rs12190287 C/G and rs12524865 C/A (Table S1) were annealed at 95 degrees for 10 minutes in annealing buffer and allowed to cool to room temperature. Double-stranded DNA fragments were then subcloned into the MCS of the minimal promoter containing pLuc-MCS vector (Agilent). Constructs were validated by Sanger sequencing. Empty vector (pLuc-MCS), rs12190287-C or rs12190287-G and *Renilla* luciferase constructs were transfected into HCASMC, RASMC, A7r5, HepG2, and HEK using Lipofectamine 2000. Media was changed after 6 hours, and dual luciferase activity was measured after 24 hours using a SpectraMax L luminometer (Molecular Devices). Relative luciferase activity (firefly/*Renilla* luciferase ratio) is expressed as the fold change of the empty vector control (pLuc-MCS).

### Electrophoretic mobility shift assay

Double stranded oligonucleotides for rs12190287-C/G, AP-1, CREB, rs12190287 mixed were obtained by annealing single stranded oligos (Table S1), as previously described [54]. Annealed oligos were then labeled with [γ^32^P]-ATP (Perkin Elmer) using T4 polynucleotide kinase (NEB) for 30 minutes at room temperature and then purified through Sephadex G-50 Quick Spin columns (Roche). After measuring radioactivity, reactions were assembled with 1× EMSA binding buffer, 1 µg poly-dIdC, 10 µg nuclear extract, 100× unlabeled probe (for competitions), 2 µg polyclonal antibody (for super-shifts), [γ^32^P]-ATP labeled probe, and incubated at room temperature for 30 min prior to protein separation on a 4% TBE gel. Gels were dried on Whatman paper using a heated vacuum drier and proteins were detected on radiographic film.

### TaqMan qPCR gene expression

Primary human coronary artery smooth muscle cells (HCASMC) were cultured in normal growth media until approximately 75% confluent, then cultured in the absence of serum and supplements for 24 hours, prior to stimulation with 50 ng/ml human recombinant PDGF-BB (R&D Systems), 5 ng/ml human recombinant TGF-β1 (R&D Systems), 100 nM PMA (Sigma) or vehicle for indicated times. Total RNA was prepared using miRNeasy Mini kit (Qiagen) and total cDNA was prepared from 0.5 µg of RNA using the TaqMan High Capacity cDNA synthesis kit (Life Technologies). TaqMan gene expression probes (Table S1) were used to amplify human *TCF21*, c-*JUN*, *JUND*, *ATF3*, and *WT1*, which were normalized to human 18S levels.

### Western blotting

Nuclear extracts were generated from HCASMC harvested at indicated time points. Protein concentrations were determined using a BCA assay (Pierce) and 50–100 µg nuclear protein for each condition was loaded on a pre-cast NuPAGE 4–12% Bis-Tris polyacrylamide gel (Invitrogen/Life Technologies), with gel run at 150 v for 1 h using MES buffer (Invitrogen/Life Technologies), and transferred to PVDF membrane at 35 v for 1 h. Membranes were blocked in 5% non-fat dry milk in 1× TBST for 1 h and incubated overnight with rabbit polyclonal antibodies against TCF21 (Sigma; 0.25 µg/ml), cJUN (Santa Cruz; 1.0 µg/ml), JUND (Santa Cruz; 1.0 µg/ml), ATF3 (Santa Cruz; 1.0 µg/ml), or WT1 (Santa Cruz; 1.0 µg/ml), followed by incubation in a secondary anti-rabbit HRP-conjugated antibody (Invitrogen/Life Technologies; 0.2 µg/ml) and detection by standard ECL (Pierce). Blots were reprobed with a mouse monoclonal antibody against GAPDH as a loading control.

### Chromatin immunoprecipitation

Chromatin immunoprecipitation (ChIP) was performed according to the Millipore EZ-ChIP protocol with slight modifications. HCASMC were cultured as described above and treated with PDGF-BB or vehicle. Cells were fixed in 1% formaldehyde to cross-link chromatin, followed by quenching with glycine. 2×10^7^ cells per condition were collected, and nuclear lysates were prepared as previously described [55]. Cross-linked chromatin nuclear extracts were sheared into ∼500 bp fragments using a Bioruptor (Diagenode) and clarified via centrifugation. 1×10^6^ nuclei per condition were precleared with 20 ul Protein G Dynabeads (Invitrogen) for 1 hour, followed by incubation with 2 ug Rabbit IgG or anti-c-Jun, JunB, JunD, ATF3, WT1 (Santa Cruz or Active Motif), H3K4Me1, H3K4Me3, H3K27Ac, H3K27Me1 (Diagenode or Abcam) overnight at 4C. Immunoprecipitated chromatin samples were incubated with 20 µl Protein G Dynabeads for 1 hour at 4C to capture the protein-DNA complexes. Complexes were washed and eluted as described. Protein-DNA cross-links were reversed, treated with RNase A and proteinase K and free DNA was purified using Qiagen PCR purification kits. Total enrichment was measured using rs12190287 or rs12524865 specific primers, or a known AP-1 regulatory region, or a negative control region using the primers listed (Table S1). Semi-quantitative PCR was used to verify ChIP products via gel electrophoresis. Quantitative real-time PCR (ViiA 7, Life Technologies) was performed using SYBR Green (Applied Biosystems) assays and fold enrichment was calculated by measuring the delta Ct – delta Ct IgG. Melting curve analysis was also performed for each ChIP primer pair.

### ChIP-sequencing

1×10^7^ HCASMCs per condition were processed as previously described [56], using anti-H3K4me1 (pAb-037-050, Diagenode), anti-H3K4me3 (pAb-003-050, Diagenode), anti-H3K27me3 (pAb-069-050, Diagenode), or anti-rabbit IgG (X 0903, DAKO). ChIP-seq library generation, cluster formation and next-generation sequencing was performed at the Stanford Functional Genomics Facility, Stanford University, Stanford CA, USA, on an Illumina MiSeq instrument. 36 bp single reads from next-generation sequencing of ChIP libraries were then mapped to the reference genome using Burrows-Wheeler Aligner (BWA). BigWig files were created using the R/Bioconductor environment.

### FAIRE-sequencing

1×10^7^ HCASMCs were mainly processed similar to the ChIP-seq samples. However, instead of preclearing and immunoprecipitation, protein-depleted DNA was extracted from cross-linked nuclear lysates by phenol-chloroform extraction. After DNA precipitation, purification and reverse cross-linking, samples were sequenced and further processed as described above.

### PCR fragment-based genotyping

Genomic DNA was isolated from >10^6^ HCASMC cultured in complete media for approximately 48 hours, using the Blood and Tissue DNA isolation Kit (Qiagen). 50 ng of gDNA template was amplified using primers flanking rs12190287 to generate 250 bp fragments. Fragments were then sequenced via Sanger sequencing using an internal forward sequencing primer, and genotypes were determined from chromatograms using Sequence Analyzer (Applied Biosystems).

### Allele-specific expression using TaqMan qPCR

Heterozygous genotypes were determined by Sanger sequencing, and RNA and cDNA prepared as described above. Allele-specific expression of *TCF21* at rs12190287 was determined using a pre-designed TaqMan SNP genotyping assay for rs12190287 (Table S1). Calibration of the SNP genotyping assay was determined by mixing 10 ng of HCASMC gDNA or cDNA, homozygous for each allele at the following ratios: 8∶1, 4∶1, 2∶1 1∶1, 1∶2, 1∶4, 1∶8. The Log2 ratio of the VIC/FAM intensity at cycle 40 was then plotted against the Log ratio of the two alleles to generate a linear regression standard curve. The Log ratio of the intensity of the two alleles from cDNA samples was fitted to the standard curve. These values were then normalized to the ratio of gDNA for each allele to obtain the normalized allelic ratio.

### Allele-specific ChIP-qPCR

Heterozygous genotypes were determined as described above. Briefly, heterozygous HCASMC were cultured for the indicated timepoints, and chromatin cross-linked, sheared and immunoprecipitated as described above. Purified DNA was then amplified using TaqMan SNP genotyping assay probes for rs12190287. The Log2 ratio of VIC/FAM intensity at cycle 40 was then fitted to the standard curve and normalized to gDNA ratio, with the normalized allelic ratio of IgG control enrichment arbitrarily set to 1.

### Allele-specific ChIP using pyrosequencing

Pyrosequencing assay for rs12190287 was generated using PyroMark Assay Design software (Qiagen). Forward rs12190287 PCR primer: 5′- and biotinylated reverse PCR primer, and forward pyrosequencing primers were synthesized by the Protein And Nucleic acid (PAN) facility (Stanford). Approximately 20 ng ChIP DNA was amplified using forward and reverse pyrosequencing primers under the following conditions: 94 C 4 min, (94 C 30 s, 60 C 30 s, 72 C 45 s) ×45, 72 C 6 min. Pyrosequencing reaction was performed on a PyroMark Q24 according to manufacturer's instructions. Allelic quantitation was obtained automatically from the mean allele frequencies derived from the peak heights using PyroMark Q24 software.

### *In silico* bioinformatics

Transcription factor binding site (TFBS) prediction was determined using the following online bioinformatics tools: TRANSFAC (BIOBASE), PROMO, MatInspector, JASPAR, and TFSearch. Sequences from dbSNP for each allele were scanned for TFBS in vertebrates meeting a minimum similarity score of 0.85. Regional association plot was generated from CARDIoGRAM meta-analysis dataset at *TCF21* using LocusZoom [57]. Linkage disequilibrium plots and haplotype frequencies were generated from Europeans in the ADVANCE cohort (Stanford) from the CARDIoGRAM consortium, and East Asians from the HALST cohort within the TAICHI consortium. Briefly, genotyping data was extracted for each region of interest using PLINK [58] and transposed files were imported into Haploview [59].

### Statistical analysis

Experiments were performed using at least three independent preparations with individual treatments/conditions performed in triplicate. Data is presented as mean ± standard deviation (SD) of replicates. GraphPad Prism 6.0 was used for statistical analysis. Comparisons between two groups were performed using paired two-tailed *t*-test. *P* values <0.05 were considered statistically significant. For multiple comparison testing, two-way analysis of variance (ANOVA) accompanied by Tukey's post-hoc test were used as appropriate.

### Ethics statement

All samples reported in this study were obtained under written informed consent for participation in the Atherosclerotic Disease, VAscular functioN, and genetiC Epidemiology (ADVANCE) and Healthy Aging Longitudinal Study in Taiwan (HALST) studies with the approval of the Institutional Review Boards of Stanford University and National Health Research Institutes, respectively.

## Supporting Information

Figure S1 Histone modification ChIP-seq and FAIRE-seq regulatory analysis in HCASMC at *TCF21* locus. (a) Representative tracks for histone modification ChIP-seq analysis to identify regulatory elements at *TCF21* locus using serum fed HCASMC (identified as homozygous for risk allele at rs12190287). H3K4me1 (marks enhancer elements), H3K4me3 (marks promoter elements), and H3K27me1 (marks repressor elements). FAIRE-seq (Formaldehyde-Assisted Isolation of Regulatory Elements) identifies regions of open chromatin, which mostly overlaps DNaseI hypersensitivity clusters shown. Green box surrounds peaks overlapping rs12190287. Similar results were observed from three independent experiments. (b) Close-up view of regulatory elements at rs12190287. These results indicate that rs12190287 resides in both an open/active chromatin region resembling an enhancer element in HCASMC.
(TIFF)

Figure S2 Distinct AP-1-like transcriptional regulatory element at rs12190287. (a) TF binding motif predicted at rs12190287, generated from positional weight matrices from TRANSFAC databases. (b) Dual-luciferase assay of rs12190287 C/G enhancer transfected in A7r5 and treated with adenylyl cyclase activator, forskolin (Fsk) or PKC activator, phorbol-12-myristate-13-acetate (PMA) and compared to consensus CRE and AP-1 reporters. Relative luciferase activities measured after 24 hours. (c) EMSA showing different protein binding complexes to rs12190287 and consensus AP-1 binding sites. Arrows and bar-headed lines represent specific and non-specific shifted complexes, respectively. Dotted arrow represents unique AP-1 shifted complex, not observed at rs12190287. Values are mean ± SD from triplicates. Similar results were observed from three independent experiments.
(TIFF)

Figure S3 Effects of AP-1 and WT1 siRNA on protein levels in HCASMC. Western blot results depicting relative knockdown of endogenous (a) c-JUN (b) JUND (c) ATF3 and (d) WT1 protein expression at 48 h in nuclear extracts from HCASMC transfected with negative control siRNA (Con si) or two distinct siRNAs against c-JUN, JUND, ATF3 or WT1, respectively. GAPDH protein levels were measured as a loading control.
(TIFF)

Figure S4 Effects of PMA or PDGF-BB treatment on TCF21 protein levels in HCASMC. Western blot results depicting changes in endogenous TCF21 protein expression levels in nuclear extracts from HCASMC treated with PMA (or DMSO vehicle control) or PDGF-BB (or vehicle control) for 3 or 6 h, respectively. GAPDH protein levels were measured as a loading control.
(TIFF)

Figure S5 Validation of allele-specific ChIP and qPCR assays at rs12192087. (a) Representative VIC and FAM fluorescence intensity traces from heterozygous samples generated from mixed homozygous HCASMC gDNA samples at indicated ratios demonstrate allelic discrimination with the TaqMan based assay. (b) Representative linear regression curve generated from the Log ratio of the VIC/FAM intensity for each Log VIC/FAM allele ratio. Raw intensity values of subsequent assays were normalized using an equation as shown to account for inherent allelic bias with the TaqMan based assay. (c) Representative pyrograms from haploChIP products sequenced using pyrosequencing primers, which resulted in comparable allelic ratios for each condition. Values are mean ± SD from triplicates. Similar results were observed from three independent experiments.
(TIFF)

Figure S6 Allele-specific transcriptional regulation at rs12524865 *in vitro*. (a) Haplotype block containing rs12524865 generated from Metabochip genotyped East Asians from HALST cohort in TAICHI study. R^2^ values relative to rs12524865 shown as percentages in parentheses. Allele frequencies for haplotypes shown to the right. Risk and protective alleles determined from CARDIoGRAM phenotypic data. Note: Only TCF21 eSNPs within the same haplotype block are shown. (b) TF binding motif predicted at rs12524865, generated from positional weight matrices from TRANSFAC databases. (c) Dual-luciferase assay of rs12524865 C/A putative enhancer co-transfected with empty vector, or dominant negative mutants for ΔCREB, ΔJun, or ΔATF3 in A7r5 and relative luciferase activities measured after 24 hours. Values represent mean ± SD from triplicates.
(TIFF)

Table S1 Oligonucleotide sequences. Custom oligonucleotide sequences are shown for the various assays. Alternatively, assay ID numbers are shown for predesigned TaqMan qPCR gene expression or genotyping probes.
(DOCX)
