# Supplementary figures and images for: Disease-Related Growth Factor and Embryonic Signaling Pathways Modulate an Enhancer of TCF21 Expression at the 6q23.2 Coronary Heart Disease Locus
Source: PLoS Genet. 2013 Jul 18;9(7):e1003652. doi: 10.1371/journal.pgen.1003652 (PMC3715442; doi:10.1371/journal.pgen.1003652)

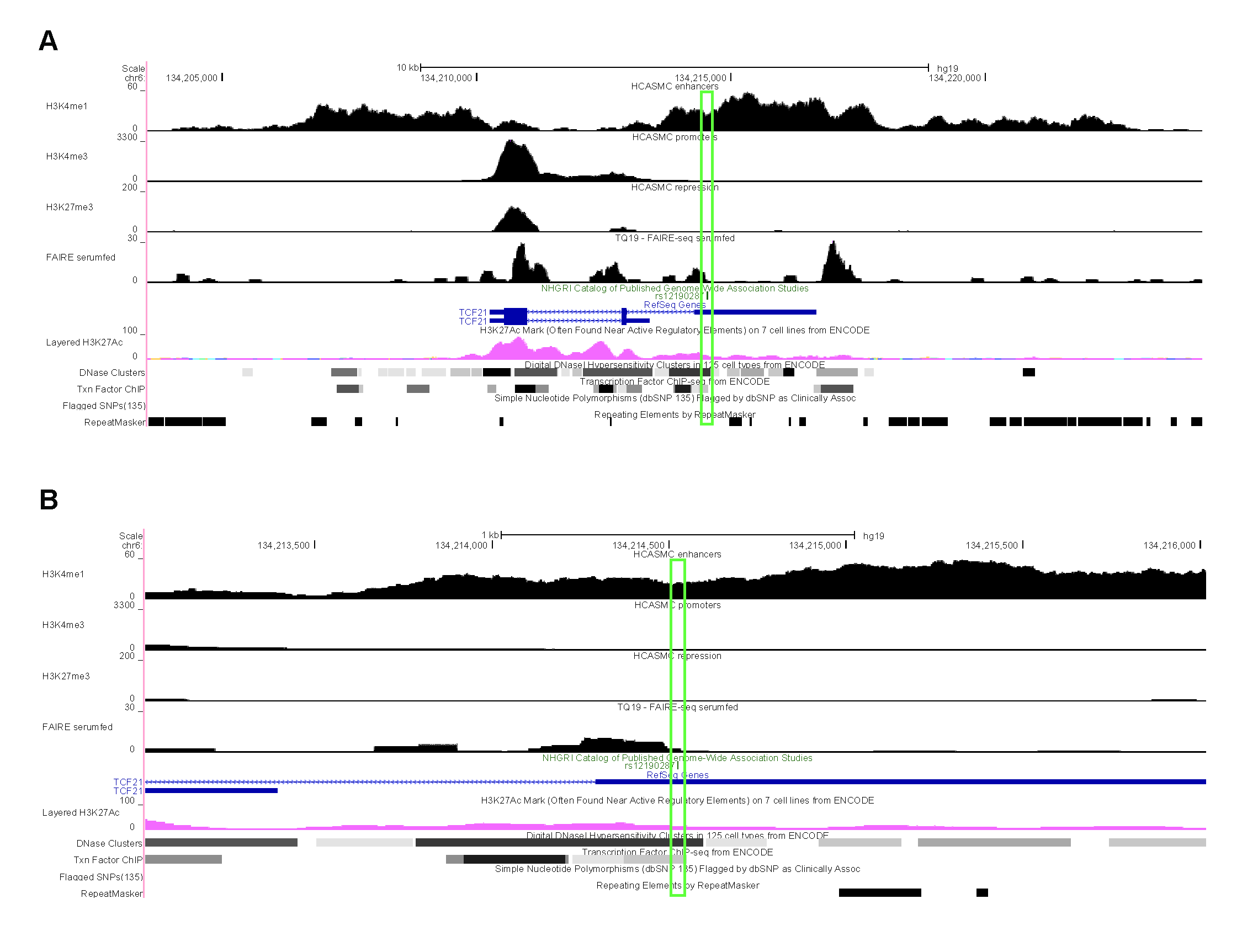

Supplement: Figure S1 — Histone modification ChIP-seq and FAIRE-seq regulatory analysis in HCASMC at TCF21 locus. (a) Representative tracks for histone modification ChIP-seq analysis to identify regulatory elements at TCF21 locus using serum fed HCASMC (identified as homozygous for risk allele at rs12190287). H3K4me1 (marks enhancer elements), H3K4me3 (marks promoter elements), and H3K27me1 (marks repressor elements). FAIRE-seq (Formaldehyde-Assisted Isolation of Regulatory Elements) identifies regions of open chromatin, which mostly overlaps DNaseI hypersensitivity clusters shown. Green box surrounds peaks overlapping rs12190287. Similar results were observed from three independent experiments. (b) Close-up view of regulatory elements at rs12190287. These results indicate that rs12190287 resides in both an open/active chromatin region resembling an enhancer element in HCASMC. (TIFF) [file pgen.1003652.s001.tiff]

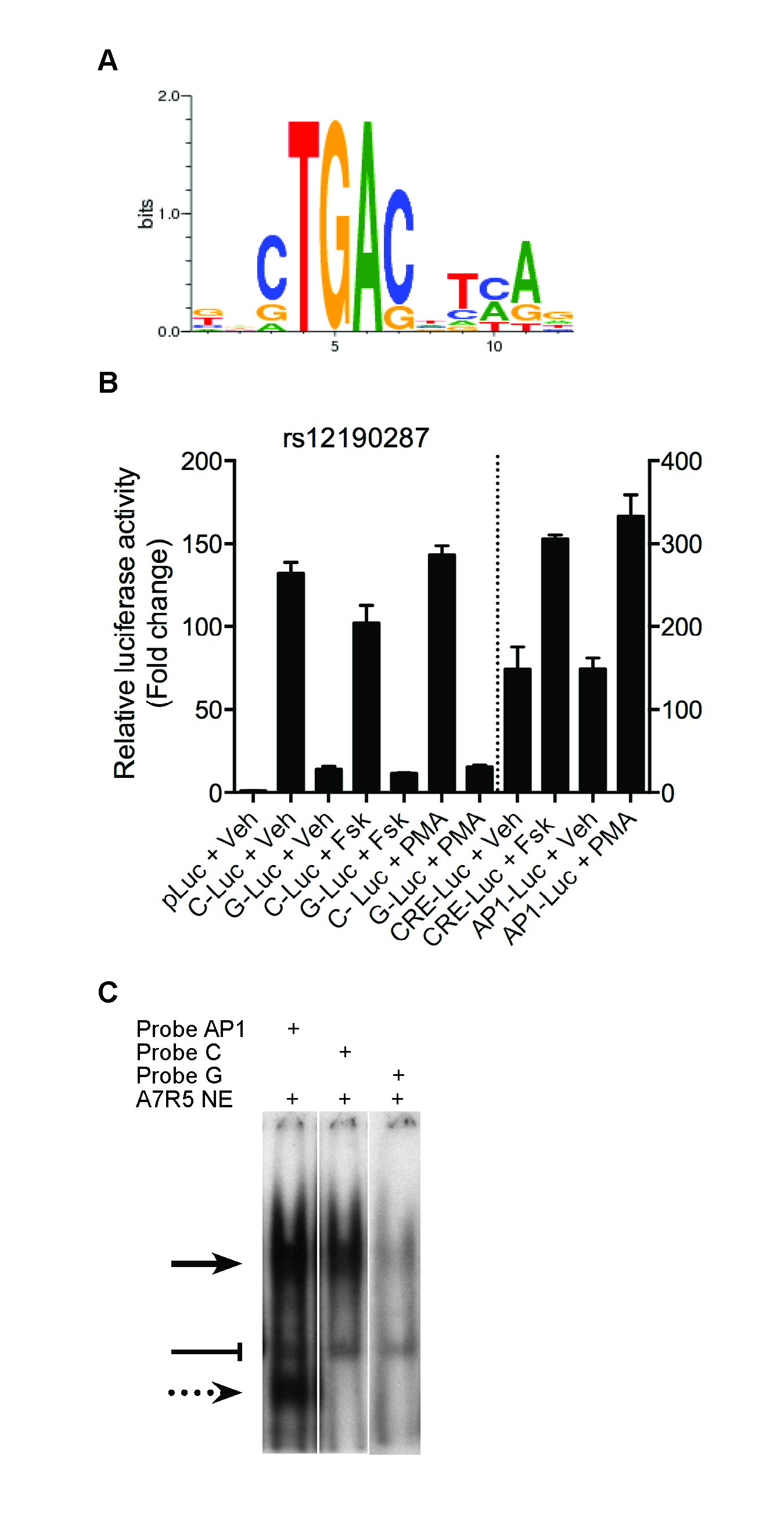

Supplement: Figure S2 — Distinct AP-1-like transcriptional regulatory element at rs12190287. (a) TF binding motif predicted at rs12190287, generated from positional weight matrices from TRANSFAC databases. (b) Dual-luciferase assay of rs12190287 C/G enhancer transfected in A7r5 and treated with adenylyl cyclase activator, forskolin (Fsk) or PKC activator, phorbol-12-myristate-13-acetate (PMA) and compared to consensus CRE and AP-1 reporters. Relative luciferase activities measured after 24 hours. (c) EMSA showing different protein binding complexes to rs12190287 and consensus AP-1 binding sites. Arrows and bar-headed lines represent specific and non-specific shifted complexes, respectively. Dotted arrow represents unique AP-1 shifted complex, not observed at rs12190287. Values are mean ± SD from triplicates. Similar results were observed from three independent experiments. (TIFF) [file pgen.1003652.s002.tiff]

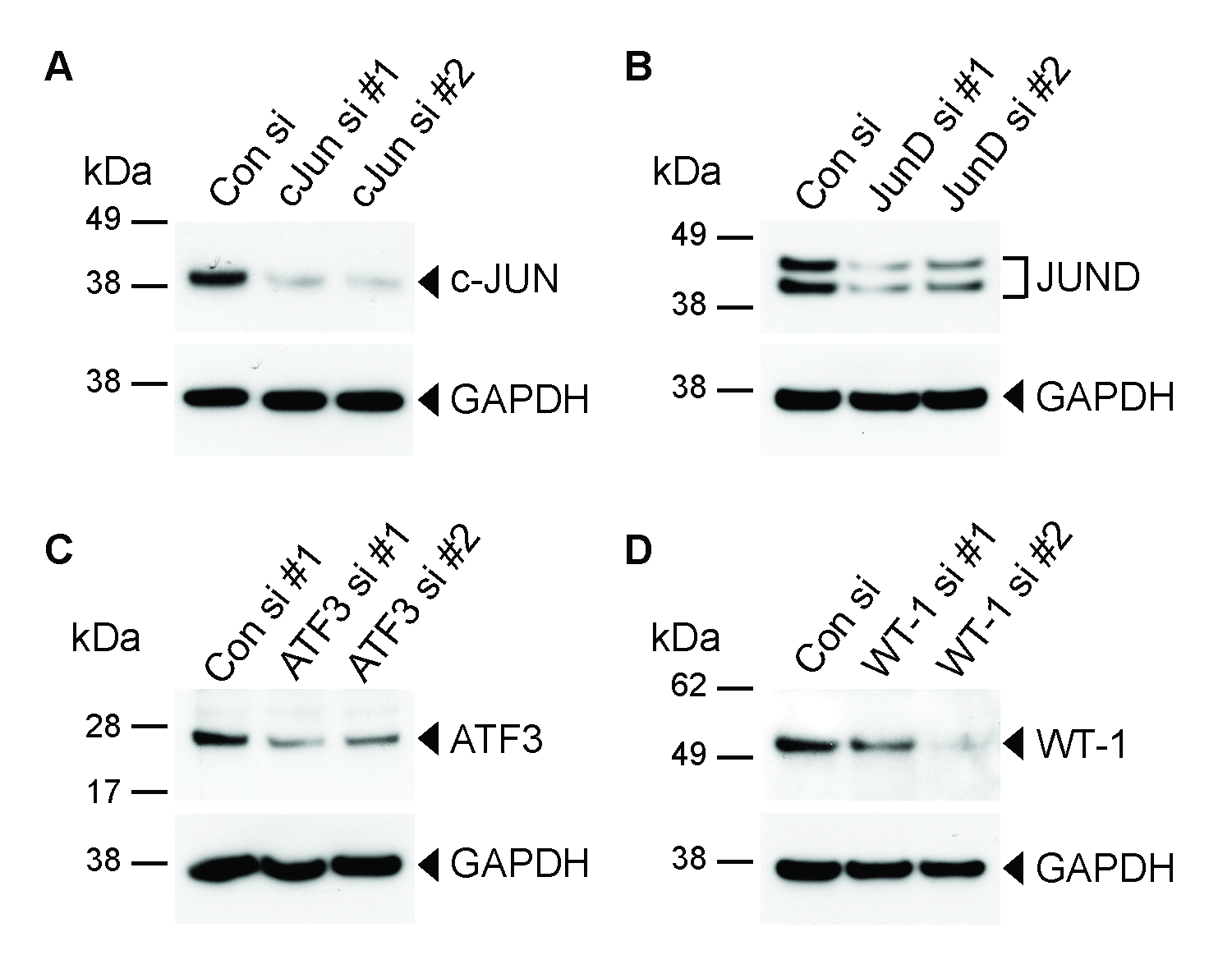

Supplement: Figure S3 — Effects of AP-1 and WT1 siRNA on protein levels in HCASMC. Western blot results depicting relative knockdown of endogenous (a) c-JUN (b) JUND (c) ATF3 and (d) WT1 protein expression at 48 h in nuclear extracts from HCASMC transfected with negative control siRNA (Con si) or two distinct siRNAs against c-JUN, JUND, ATF3 or WT1, respectively. GAPDH protein levels were measured as a loading control. (TIFF) [file pgen.1003652.s003.tiff]

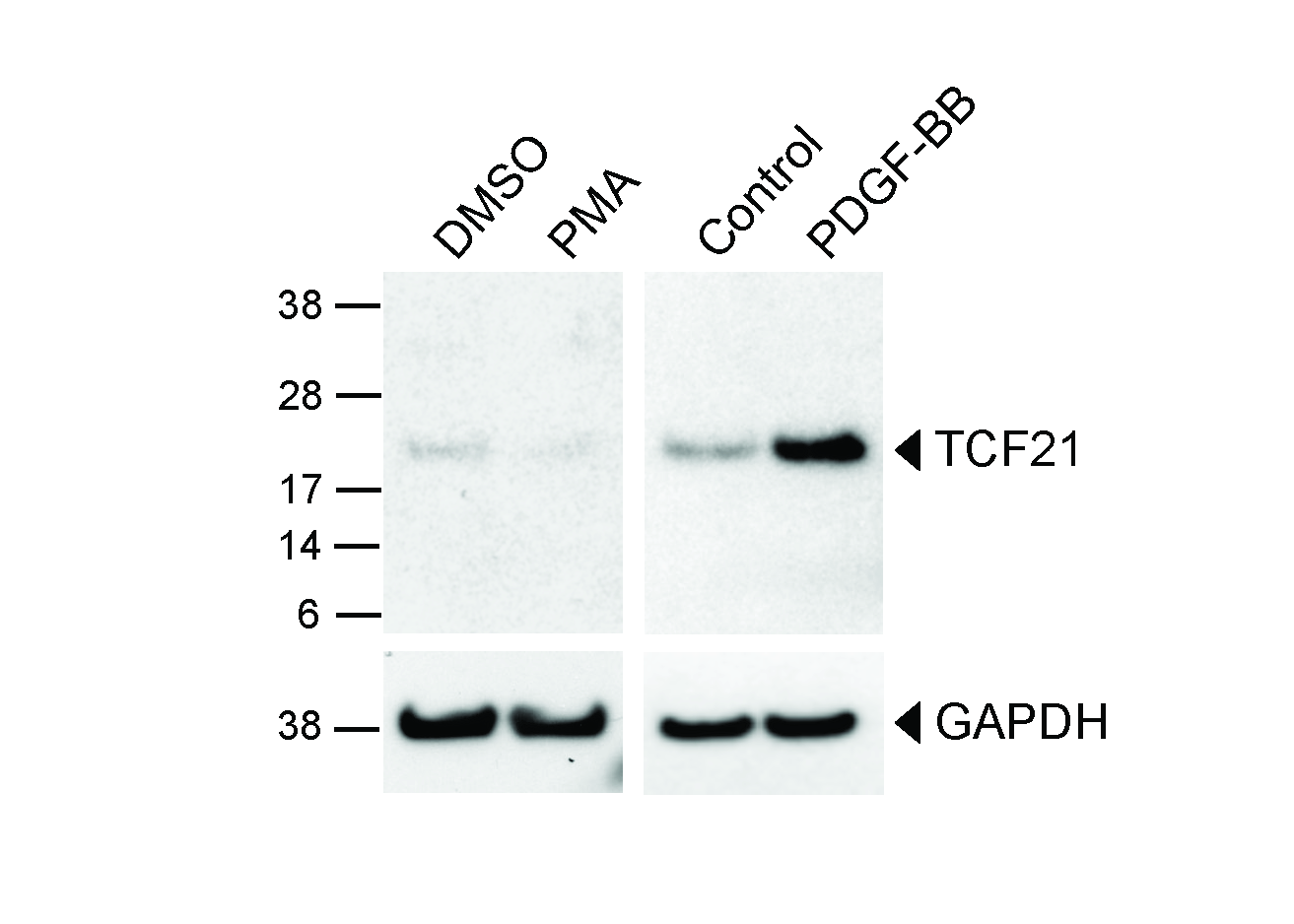

Supplement: Figure S4 — Effects of PMA or PDGF-BB treatment on TCF21 protein levels in HCASMC. Western blot results depicting changes in endogenous TCF21 protein expression levels in nuclear extracts from HCASMC treated with PMA (or DMSO vehicle control) or PDGF-BB (or vehicle control) for 3 or 6 h, respectively. GAPDH protein levels were measured as a loading control. (TIFF) [file pgen.1003652.s004.tiff]

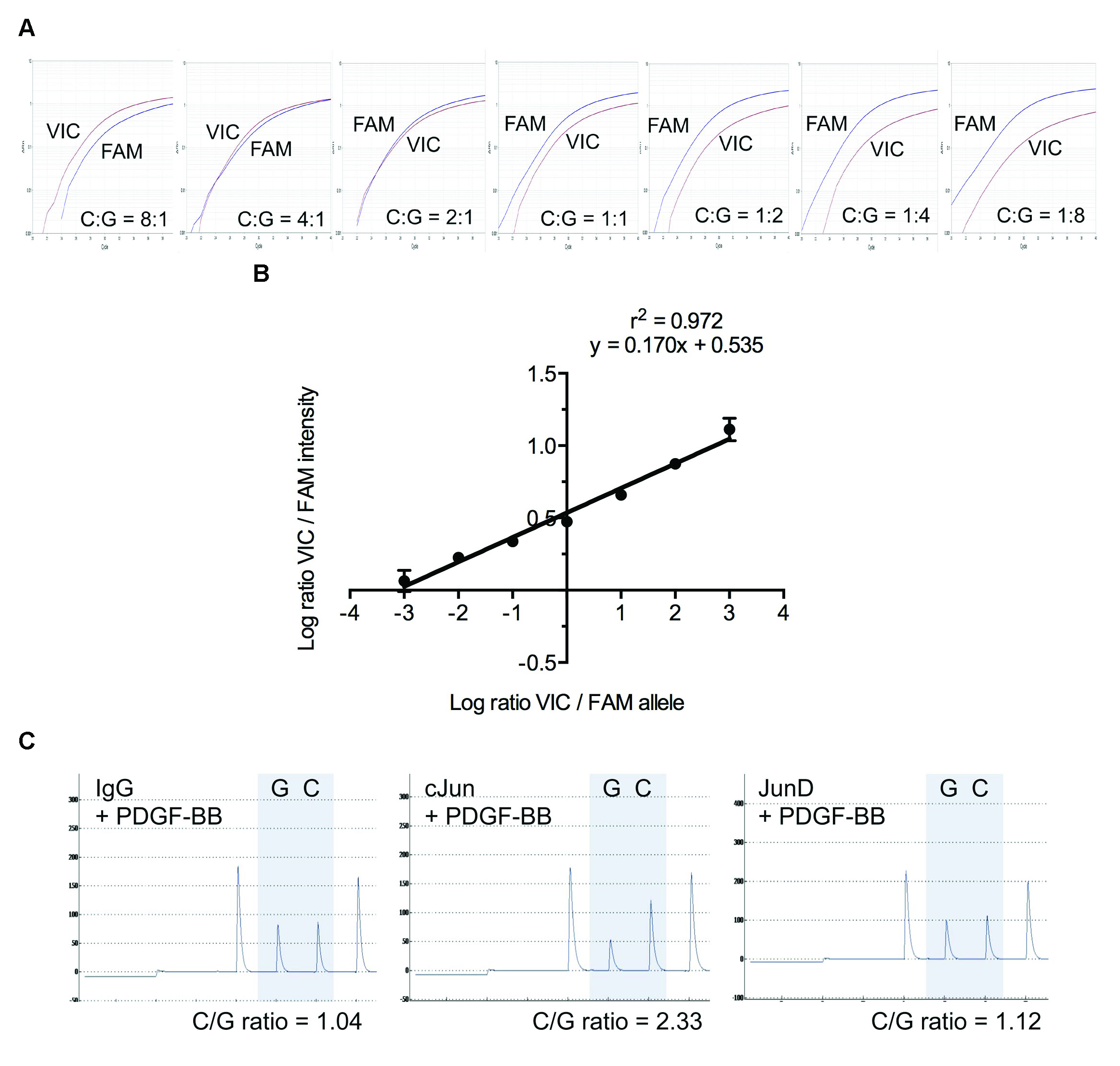

Supplement: Figure S5 — Validation of allele-specific ChIP and qPCR assays at rs12192087. (a) Representative VIC and FAM fluorescence intensity traces from heterozygous samples generated from mixed homozygous HCASMC gDNA samples at indicated ratios demonstrate allelic discrimination with the TaqMan based assay. (b) Representative linear regression curve generated from the Log ratio of the VIC/FAM intensity for each Log VIC/FAM allele ratio. Raw intensity values of subsequent assays were normalized using an equation as shown to account for inherent allelic bias with the TaqMan based assay. (c) Representative pyrograms from haploChIP products sequenced using pyrosequencing primers, which resulted in comparable allelic ratios for each condition. Values are mean ± SD from triplicates. Similar results were observed from three independent experiments. (TIFF) [file pgen.1003652.s005.tiff]

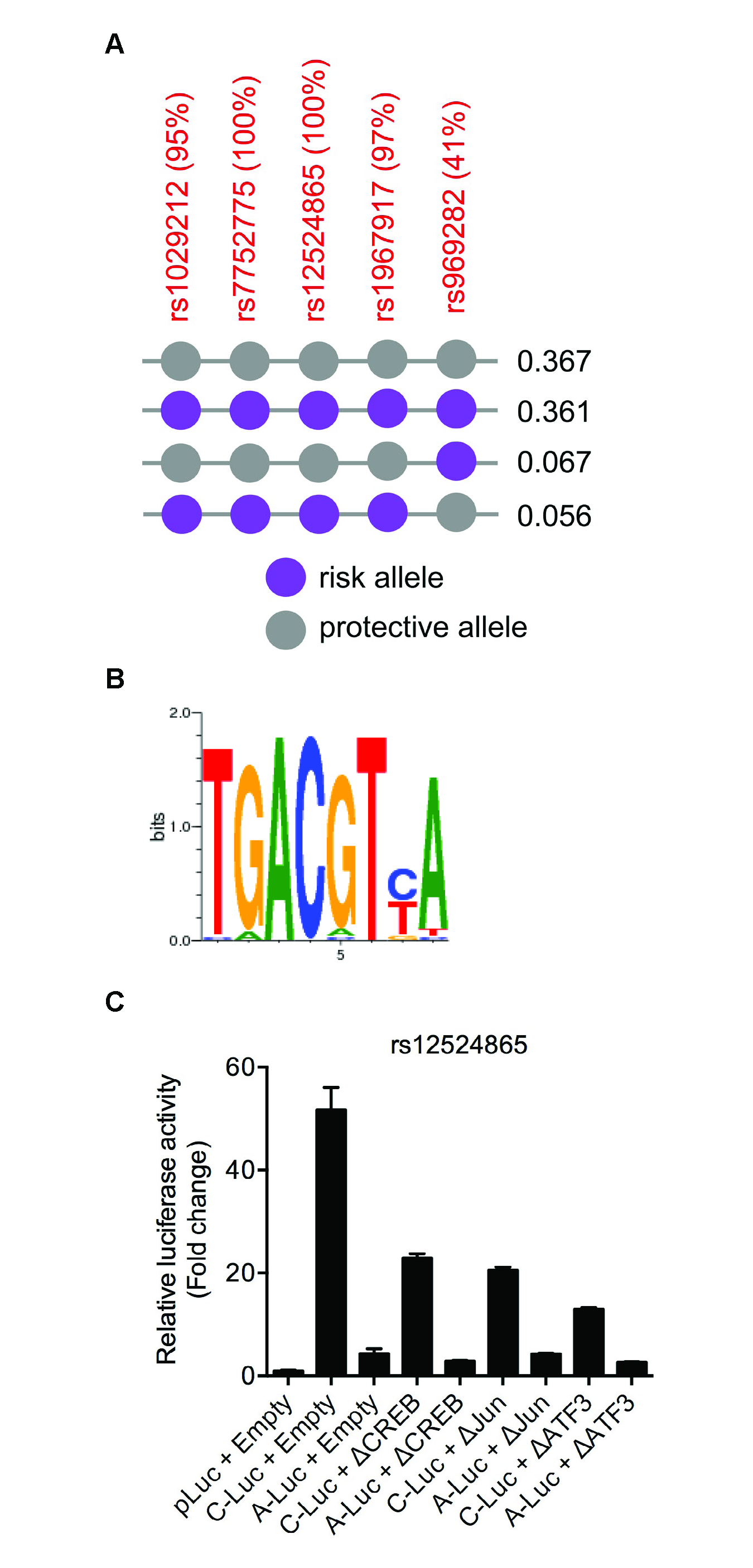

Supplement: Figure S6 — Allele-specific transcriptional regulation at rs12524865 in vitro. (a) Haplotype block containing rs12524865 generated from Metabochip genotyped East Asians from HALST cohort in TAICHI study. R2 values relative to rs12524865 shown as percentages in parentheses. Allele frequencies for haplotypes shown to the right. Risk and protective alleles determined from CARDIoGRAM phenotypic data. Note: Only TCF21 eSNPs within the same haplotype block are shown. (b) TF binding motif predicted at rs12524865, generated from positional weight matrices from TRANSFAC databases. (c) Dual-luciferase assay of rs12524865 C/A putative enhancer co-transfected with empty vector, or dominant negative mutants for ΔCREB, ΔJun, or ΔATF3 in A7r5 and relative luciferase activities measured after 24 hours. Values represent mean ± SD from triplicates. (TIFF) [file pgen.1003652.s006.tiff]
