# Supplementary material for: Disease-Related Growth Factor and Embryonic Signaling Pathways Modulate an Enhancer of TCF21 Expression at the 6q23.2 Coronary Heart Disease Locus
Source: PLoS Genet. 2013 Jul 18;9(7):e1003652. doi: 10.1371/journal.pgen.1003652 (PMC3715442; doi:10.1371/journal.pgen.1003652)
Supplement: Table S1 — Oligonucleotide sequences. Custom oligonucleotide sequences are shown for the various assays. Alternatively, assay ID numbers are shown for predesigned TaqMan qPCR gene expression or genotyping probes. (DOCX) [file pgen.1003652.s007.docx]

**Table S1. Oligonucleotide sequences**

| **Assay** | **Oligonucleotide** | **Sequence (5’-3’) or Assay ID** |
| --- | --- | --- |
| Luciferase reporter | rs12190287-4X-C For | AGCT(TCGGTGACTTCATCC)4x |
|  | rs12190287-4X-C Rev | GATC(GGATGAAGTCACCGA)4x |
|  | rs12190287-4X-G For | AGCT(TCGGTGAGTTCATCC)4x |
|  | rs12190287-4X-G Rev | GATC(GGATGAACTCACCGA)4x |
|  | rs12190287-DelT-4X-C For | AGCT(TCGGTGACTCATCC)4x |
|  | rs12190287-DelT-4X-C Rev | GATC(GGATGAGTCACCGA)4x |
|  | rs12190287-DelT-4X-G For | AGCT(TCGGTGAGTCATCC)4x |
|  | rs12190287-DelT-4X-G Rev | GATC(GGATGACTCACCGA)4x |
|  | rs12190287-T/A-4X-C For | AGCT(TCGGTGACATCATCC)4x |
|  | rs12190287-T/A-4X-C Rev | GATC(GGATGATGTCACCGA)4x |
|  | rs12190287-T/A-4X-G For | AGCT(TCGGTGAGATCATCC)4x |
|  | rs12190287-T/A-4X-G Rev | GATC(GGATGATCTCACCGA)4x |
|  | rs12524865-4X-A For | AGCT(AACTTAAAGTCAGTC)4x |
|  | rs12524865-4X-A Rev | GATC(GACTGACTTTAAGTT)4x |
|  | rs12524865-4X-C For | AGCT(AACTTAACGTCAGTC)4x |
|  | rs12524865-4X-C Rev | GATC(GACTGACGTTAAGTT)4x |
| EMSA | rs12190287-C For | ACTTCGGTGACTTCATCCACC |
|  | rs12190287-C Rev | GGTGGATGAAGTCACCGAAGT |
|  | rs12190287-G For | ACTTCGGTGAGTTCATCCACC |
|  | rs12190287-G Rev | GGTGGATGAACTCACCGAAGT |
|  | AP1 For | CCTTGGGGTGACATCATGGGCTAT |
|  | AP1 Rev | ATAGCCCATGATGTCACCCCAAGG |
|  | CREB For | AGAGATTGCCTGACGTCAGAGAGCTAG |
|  | CREB Rev | CTAGCTCTCTGACGTCAGGCAATCTCT |
|  | rs121-C mixed For (Neg control) | ACTTCGGTGACGTCATCCACC |
|  | rs121-C mixed Rev (Neg control) | GGTGGATGACGTCACCGAAGT |
| ChIP | rs12190287 ChIP For | CGACCACATTACCAAGCGCAATTC |
|  | rs12190287 ChIP Rev | TGGAAGGGTATCCTGACATCTTGA |
|  | rs12524865 ChIP For | CTTGCTAACCAACACTGAAA |
|  | rs12524865 ChIP Rev | GCCAGAAATAGTAACCTGTTG |
|  | MYOG ChIP For (Neg control) | CCTTGATGTGCAGCAACAGC |
|  | MYOG ChIP Rev (Neg control) | CCAACGCCACAGAAACCTG |
|  | FOSB ChIP For (Pos control) | TCATGCAAGTGACCAGATCGA |
|  | FOSB ChIP Rev (Pos control) | GCACTGTCCAGCAAGAGGTCT |
| SNP Genotyping | rs12190287 PCR For | GCTCTCTGTCAGCAATAGTGACCT |
|  | rs12190287 PCR Rev | TGTGTTCTTGAAAGTCAAGTTGGG |
|  | rs12190287 Seq For | ACCACATTACCAAGCGCAATTCCC |
|  | rs12190287 Seq Rev | ATGGAAGGGTATCCTGACATCTTG |
|  | rs12190287 PCR 1 For | GCTCTCTGTCAGCAATAGTGACCT |
|  | rs12190287 PCR 1 Rev | TGTGTTCTTGAAAGTCAAGTTGGG |
|  | rs12190287 Seq 1 For | ACCACATTACCAAGCGCAATTCCC |
|  | rs12190287 Seq 1 Rev | ATGGAAGGGTATCCTGACATCTTG |
|  | rs12190287 PCR 2 For | AGGCAGATCCTGGCTAACGACAAA |
|  | rs12190287 PCR 2 Rev | TGAATTTGAGGAACGGGACTCTGG |
|  | rs12190287 Seq 2 For | TGACCTTGGAGTTTGGTACCTGGA |
| Pyrosequencing | rs12190287 Pyro 1 For | Biotin-CCACATTACCAAGCGCAATT |
|  | rs12190287 Pyro 1 For | TGGGGTGATGGAAGGGTAT |
|  | rs12190287 Pyro Seq | ATAGACAGGTGGATGA |
| TaqMan SNP qPCR | rs12190287 | C_32243431_10 |
|  | rs12524865 | C_32243437_10 |
| TaqMan qPCR | Human TCF21_1/2 | Hs00162646_m1 |
|  | Human TCF21_2 | Hs04230774_s1 |
|  | Human TCF21_1 | Hs01546814_m1 |
|  | Human JUN | Hs01103582_s1 |
|  | Human JUND | Hs02330233_u1 |
|  | Human ATF3 | Hs00231069_m1 |
|  | Human 18S | Hs99999901_s1 |
